# microRNA-Based Network and Pathway Analysis for Neuropathic Pain in Rodent Models

**DOI:** 10.3389/fmolb.2021.780730

**Published:** 2022-01-13

**Authors:** Yi-Li Zheng, Xuan Su, Yu-Meng Chen, Jia-Bao Guo, Ge Song, Zheng Yang, Pei-Jie Chen, Xue-Qiang Wang

**Affiliations:** ^1^ Department of Sport Rehabilitation, Shanghai University of Sport, Shanghai, China; ^2^ The Second School of Clinical Medical, Xuzhou Medical University, Xuzhou, China; ^3^ Department of Rehabilitation Medicine, Ruijin Hospital, Shanghai Jiao Tong University School of Medicine, Shanghai, China; ^4^ Department of Rehabilitation Medicine, Shanghai Jiao Tong University Affiliated Sixth People’s Hospital, Shanghai, China; ^5^ Department of Rehabilitation Medicine, Shanghai Shangti Orthopaedic Hospital, Shanghai, China

**Keywords:** bioinformatics analysis, miRNA, neuropathic pain, functional enrichment analysis, biomarker

## Abstract

Neuropathic pain (NP) is poorly managed, and in-depth mechanisms of gene transcriptome alterations in NP pathogenesis are not yet fully understood. To determine microRNA-related molecular mechanisms of NP and their transcriptional regulation in NP, PubMed, Embase, Web of Science and CINAHL Complete (EBSCO) were searched from inception to April 2021. Commonly dysregulated miRNAs in NP were assessed. The putative targets of these miRNAs were determined using TargetScan, Funrich, Cytoscape and String database. A total of 133 literatures containing miRNA profiles studies and experimentally verify studies were included. Venn analysis, target gene prediction analysis and functional enrichment analysis indicated several miRNAs (miR-200b-3p, miR-96, miR-182, miR-183, miR-30b, miR-155 and miR-145) and their target genes involved in known relevant pathways for NP. Targets on transient receptor potential channels, voltage-gated sodium channels and voltage-gated calcium channels may be harnessed for pain relief. A further delineation of signal processing and modulation in neuronal ensembles is key to achieving therapeutic success in future studies.

## Introduction

Neuropathic pain (NP) is poorly managed and causes by a disease or lesion of the somatosensory nervous system (Cohen and Mao, 2014), accompanied by metabolic diseases, mechanical trauma or tumor invasion; it concerns various pathophysiological changes within the peripheral and/or central nervous system (Costigan et al., 2009; Zhang Y. H et al., 2021). A distinctive feature of peripheral neuropathic pain is mechanical allodynia, which is triggered by light touch (Costigan et al., 2009; Liu et al., 2018). A number of rodent NP models that have been established to shape exploration efforts in the pathophysiological mechanisms of NP in the nervous system include the sciatic chronic constriction injury (CCI), spared nerve injury (SNI), spinal nerve ligation (SNL), sciatic nerve transection, diabetic neuropathy and drug-induced neuropathy models. Although human perception of pain is subjective that it cannot be completely duplicated in animal models, tactile allodynia in rodent models is regarded to be a corresponding pattern for neuropathic mechanical hypersensitivity in patients. These models commonly provide a clear picture of cause-effect relationship or particular biomarkers in NP.

In recent years, several studies have comprehensively discussed epigenetic modifications have dominated our understanding about activation and suppression of various gene expressions in the persistent and development of NP models (Penas and Navarro, 2018; Guo et al., 2019; An et al., 2021; Zheng et al., 2021). Unfortunately, in-depth mechanisms of gene transcriptome alterations in NP pathogenesis are not yet fully understood. Our previous study (Guo et al., 2019) have investigated the biological function of microRNAs (miRNAs) in frequently used NP rat model like CCI, SNI and SNL. Although miRNAs are non-coding RNAs containing only 19–25 nucleotides and are not directly involved in peptide synthesis, they markedly control biological processes by affecting mRNA stability, as well as protein translation (Lopez-Gonzalez et al., 2017; Song et al., 2020). Nervous tissues, such as dorsal root ganglia (DRG), spinal cord and prefrontal cortex (PFC), are of considerable concern in terms of the therapeutic potential of miRNAs in NP (Tang et al., 2021). A number of miRNAs, such as miR-183 cluster, miR-155, miR-145, miR-203 and miR-200b/429, are detected in nervous tissues and are involved in NP by affecting neuronal excitability, neuroinflammation, or neuronal plasticity (Hamada et al., 2014; Peng et al., 2017; Shi et al., 2018; Zhang Y et al., 2020). In addition, the dysregulation of certain miRNAs mediates downstream molecular mediator of pain, which contain transient receptor potential receptor channels, purinergic receptors, and voltage-gated sodium and calcium channels (Yan et al., 2020a; Yeh et al., 2020; Li et al., 2021). Although numerous studies have attempted to provide novel mechanistic insights into the role of miRNAs roles in NP, the entire pain pathway still remains to be elucidated. Hence, we performed comprehensive literature search on the microRNA-related molecular mechanisms of NP, and bioinformatics analysis of their transcriptional regulation. This study may shed light on the enigmatic pathophysiology of NP.

## Methods

### Search Strategy

A systematic search was performed in April 2021 on four electronic databases, namely, PubMed, Embase, Web of Science and CINAHL Complete (EBSCO). The terms used for search words included the following: (“neuropathic pain”, “Neuralgia”, “Nerve pain”, “sciatica”, “chronic constriction injury”, “spinal nerve ligation”, “spared nerve injury”, “chronic compression dorsal root ganglion”, “CCI”, “SNI”, “SNL” or “CCD”) and (“microRNA”, “micro-RNA”, “mir*”, or “miRNA”). No language restrictions were employed. The reference lists of all identified articles were examined. Full details of the search strategy for all databases can be found in Supplementary Appendix S1. Subsequently, all search results were imported in EndNote X7 (Thomson Research Soft, Stamford, United States). Duplicate items, reviews, abstracts and full texts were removed.

### Inclusion Criteria

Compared with our previous study (Guo et al., 2019), this study focused not only on NP rat model like CCI, SNI and SNL, but also on mouse model, meanwhile, contained drug- and disease-induced neuropathy models. So, several unambiguous inclusion criteria were listed as follows: 1) types of studies: only original articles exploring miRNAs’ roles in NP through comparison with the rodent models in NP condition and those without NP. Conference abstracts, conference presentations, book chapters, book reviews, case studies, meta-analysis, news items and corrections were excluded; 2) types of animal models: a. mouse or rat models of NP surgical models containing CCI, SNI, SNL, sciatic nerve transection, partial sciatic nerve injury, brachial plexus avulsion and trigeminal neuralgia, b. mouse or rat models of drug-induced NP models, including vincristine, cisplatin, oxaliplatin, bortezomib and taxanes, c. mouse or rat models of disease-induced NP models, including diabetes-induced neuropathy, post-herpetic neuralgia and cancer pain; 3) types of samples: nervous tissues, such as brain, spinal cord, DRG, sciatic nerve, and nervous cells, just like microglia, astrocytes and DRG neurons; and 4) types of measurements: miRNA expression measured in the way of polymerase chain reaction, microarray analysis or TaqMan low density array.

### Data Extraction

Data were extracted independently by two authors (Su X and Chen YM). For each included study, the first author’s family name, publication year, country, experimental design (e.g., experimental models, region used) and miRNA details (e.g., expression alteration, target genes and functions) were extracted. In case of disagreement, the two authors discussed the issue or consulted a third author (Wang XQ).

### Bioinformatics Analysis of miRNAs and Its Targets

Venn diagram analysis showed the number of overlapping genes by Functional Enrichment analysis tool (FunRich v3.1.3; installation package downloaded from http://www.funrich.org/). The fold value for dysregulated miRNAs in the matrix table is greater than or equal to 1.5. TargetScan (http://www.targetscan.org/) was used to predict the target genes of miRNAs and further verified the functional specificity of miRNAs. Then, protein-protein interaction (PPI) networks were established to having an integral understanding about the association among the overlapping targets of differentially expressed miRNAs. Additionally, PPI data were achieved from the String database (http://string-db.org/) and imported in Cytoscape v3.7.2 (Shannon et al., 2003) to draw a map. The high confidence score with >0.7 was defined to establish PPI network. Gene Ontology (GO) annotation was used to probe the functional roles of putative targets from three aspects, namely, biological process (BP), cellular component (CC) and molecular functions (MF). Kyoto Encyclopedia of Genes and Genomes (KEGG) analysis was conducted to predict the metabolic pathways of gene products. GO analysis and KEGG analysis were performed in DAVID (The Database for Annotation, Visualization and Integrated Discovery; https://david.ncifcrf.gov/). Significantly targeted pathways were identified by Fisher exact test with *p*-value <0.05. GraphPad Prism 8.0.1 (GraphPad Software, La Jolla California United States, http://www.graphpad.com) was used to present the enrichment results.

## Results

**FIGURE 1:**
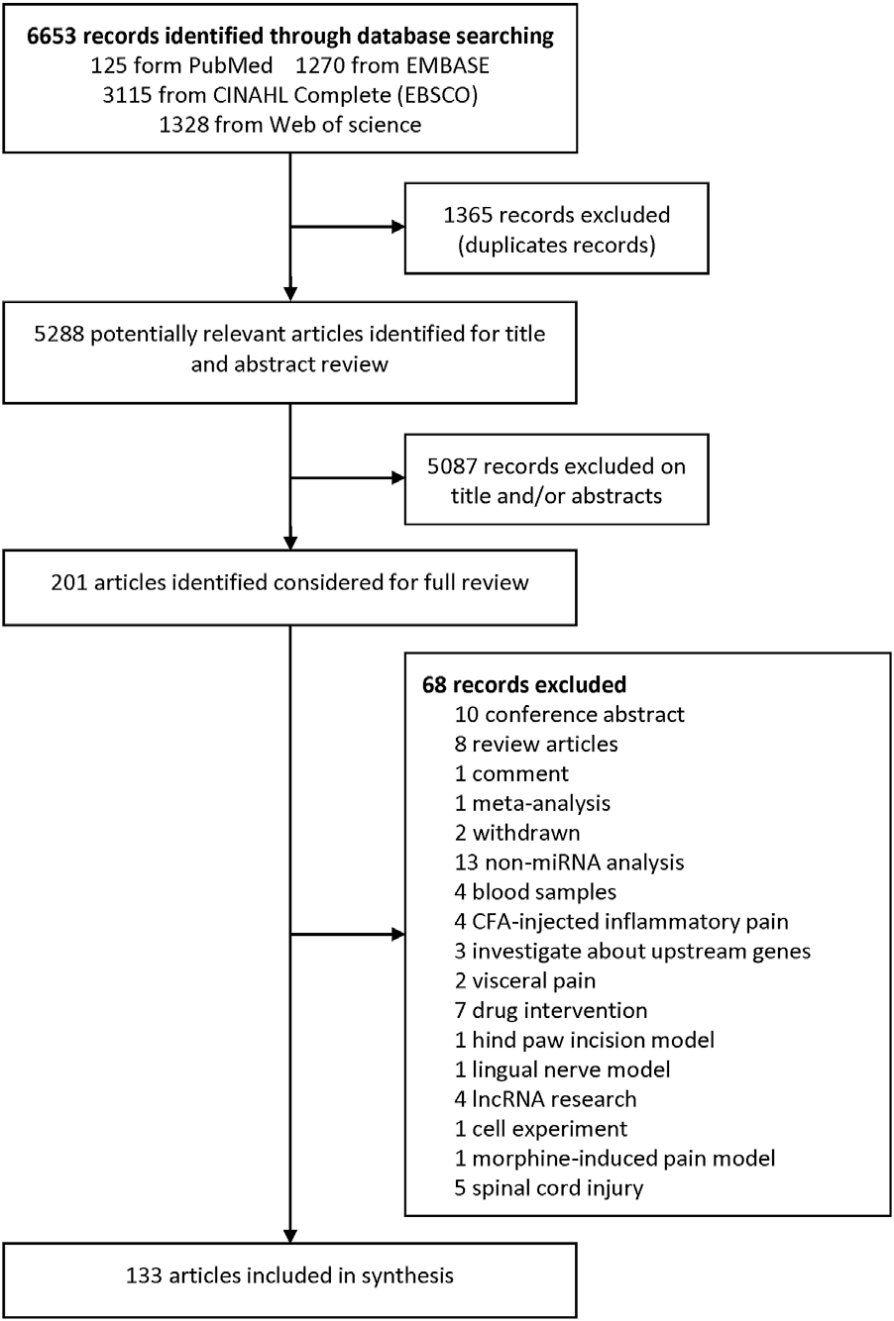
Flow chart of the study selection procedure.

We identified 6,653 records from online databases. After duplicate records were removed, 5,288 items remained. Two authors assessed the title and abstract and found that 201 articles were pushed on full-text review. At last, 133 literatures met the eligibility criteria and were included (excluded articles are listed in Supplementary Table S1). Among these included studies, 23 were about miRNA profiles (Supplementary Tables S2, S3), 113 were about miRNA experimental verification and three (Dong et al., 2014; Lu et al., 2017; Liu L et al., 2020) were both about miRNA profile and experimental verification. Among the 113 miRNA experimental verification articles, 96 are about NP surgical models (Supplementary Table S4), 6 are drug-induced NP models (Supplementary Table S5), and 11 are disease-induced NP models (Supplementary Table S6). The flowchart of the study selection procedure is shown in Figure 1.

### Study Characteristics

Details on the characteristics of articles that met inclusion criteria are shown in Supplementary Tables S2–S6. A total of 23 articles examined differential expression of miRNAs in NP rodent model’s expression profiling study (Supplementary Tables S2, S3). Among these articles, the quantity of the significantly dysregulated miRNAs ranged from 1 to 59. As of April 2021, there are no expression profiles papers on drug induced NP models, 21 expression profiles papers on NP surgical models and 2 expression profiles papers on drug induced NP models. 34.8% (8/23) of the studies collected DRGs and 43.5% (10/23) collected SDH to examine differentially expressed miRNAs between NP rats/mice and sham group rats/mice. In the latest study (Chen et al., 2021), miRNA changes in DRG after SNI were analyzed by DESeq2 and verified by qRT-PCR. Three miRNAs (miR-351-5p, miR-125a-5p and miR-125b-5p) were significantly down-regulated, whilst no up-regulated miRNA was mentioned.

A total of 113 articles experimentally verified that 91 miRNAs might take part in NP regulation (Supplementary Tables S4–S6). Specifically, 96 articles verified 79 miRNAs in NP surgical models (Supplementary Table S4), 6 articles verified 5 miRNAs in drug-induced NP models (Supplementary Table S5) and 11 articles verified 11 miRNAs in disease-induced NP models (Supplementary Table S6). The function of these NP-related miRNAs was generally categorized by neuroinflammation, neuronal adaptivity, neuronal excitability, neuronal plasticity, neuronal proliferation, DNA methylation, neuroimmune and GABAergic synapses excitability.

Supplementary Table S4 shows a comparison of the sham-operated group and five NP surgical models, namely, SNL, SNI, CCI, sciatic nerve transection, partial sciatic nerve ligation and CFA-induced prosopalgia. As for tissue analysis, miRNA expression was altered in the nervous tissues and nervous cells. Nervous tissues included DRG, spinal cord, trigeminal ganglions, caudal medulla and sciatic nerve; nervous cells included microglia, DRG neurons and astrocytes. Briefly, 61.5% (59/96) of experimental studies evaluated the miRNA expression in spinal cord, 38.4% (37/96) in DRG, 4.1% (4/96) in sciatic nerve, 2.1% (2/96) in trigeminal ganglions, 1.0% (1/96) in nucleus accumbens, 1.0% (1/96) in caudal medulla and 1.0% (1/96) in sural nerve. Moreover, the expression level of several miRNAs (Jiang et al., 2016; Tramullas et al., 2018; Wang et al., 2018; Zhang X et al., 2020; Zhou et al., 2020; Sun et al., 2021; Zhang J et al., 2021) in DRG and the spinal cord were changed. In terms of included studies, 58 miRNAs were down-regulated in the NP process, of which miR-98, miR-96, miR-182, miR-183, miR-7a, miR-30b, miR-206, miR-200b, miR-429, miR-150 and miR-145 were reported two times or more. 26 miRNAs were up-regulated, of which miR-21, miR-155 and miR-195 were reported two times or more. In the down-regulated and up-regulated miRNAs, there were five overlapping genes, namely, miR-23a, miR-142-3p, miR-21-5p, miR-15a and miR-101. Among them, miR-23a expression decreased in SDH (Pan et al., 2018) and increased in DRGs (Zhang Y et al., 2021); miR-142-3p expression decreased in DRGs (Zhang et al., 2018) and increases in the sciatic nerve (Li et al., 2021); miR-21-5p expression decreased in the dorsal spinal cords (Zhong et al., 2019) and increased in DRGs (Simeoli et al., 2017); miR-15a and miR-101 showed contradictory results in the spinal cords and SDH, respectively. Cai et al. (2020) indicated miR-15a was down-regulated in spinal cords, while Li T et al. (2019) indicated miR-15a was up-regulated. Xie et al. (2020) revealed miR-101 was down-regulated in SDH, whilst Qiu et al. (2020) disclosed miR-101 was up-regulated.

Six studies verified that five miRNAs changed in NP induced by chemotherapeutic drugs, such as paclitaxel, oxaliplatin and bortezomib (Supplementary Table S5). SDH and DRGs were collected for measurement. miR-30b-5p (Li L et al., 2019) and miR-141-5p (Zhang and Chen, 2021) were down-regulated in chemotherapeutic drug-induced NP; miR-500 (Huang et al., 2016), miR-15b (Ito et al., 2017) and miR-155 (Miao et al., 2019; Duan et al., 2020) were up-regulated. NP induced by cancer pain and diabetes are shown in Supplementary Table S6. Four studies showed that three miRNAs were down-regulated (Elramah et al., 2017; Wu X. P. et al., 2019; Liu C et al., 2020) (miR-124, miR-329 and miR-300), and 1 miRNA was up-regulated (Gandla et al., 2017) (miR-34c-5p) in NP induced by bone cancer pain; and 7 studies indicated 5 down-regulated miRNAs (Yang et al., 2017; Feng et al., 2018; Wu B. et al., 2019; Yan et al., 2020b; Wu et al., 2020) (miR-190a-5p, miR-146a, miR-193a, miR-145 and miR-590-3p) and 2 up-regulated miRNAs (Chen et al., 2019; Chang et al., 2020) (miR-155 and miR-133a-3p) in diabetic neuropathic pain.

### Target Prediction and Venn Diagram Analysis

**FIGURE 2:**
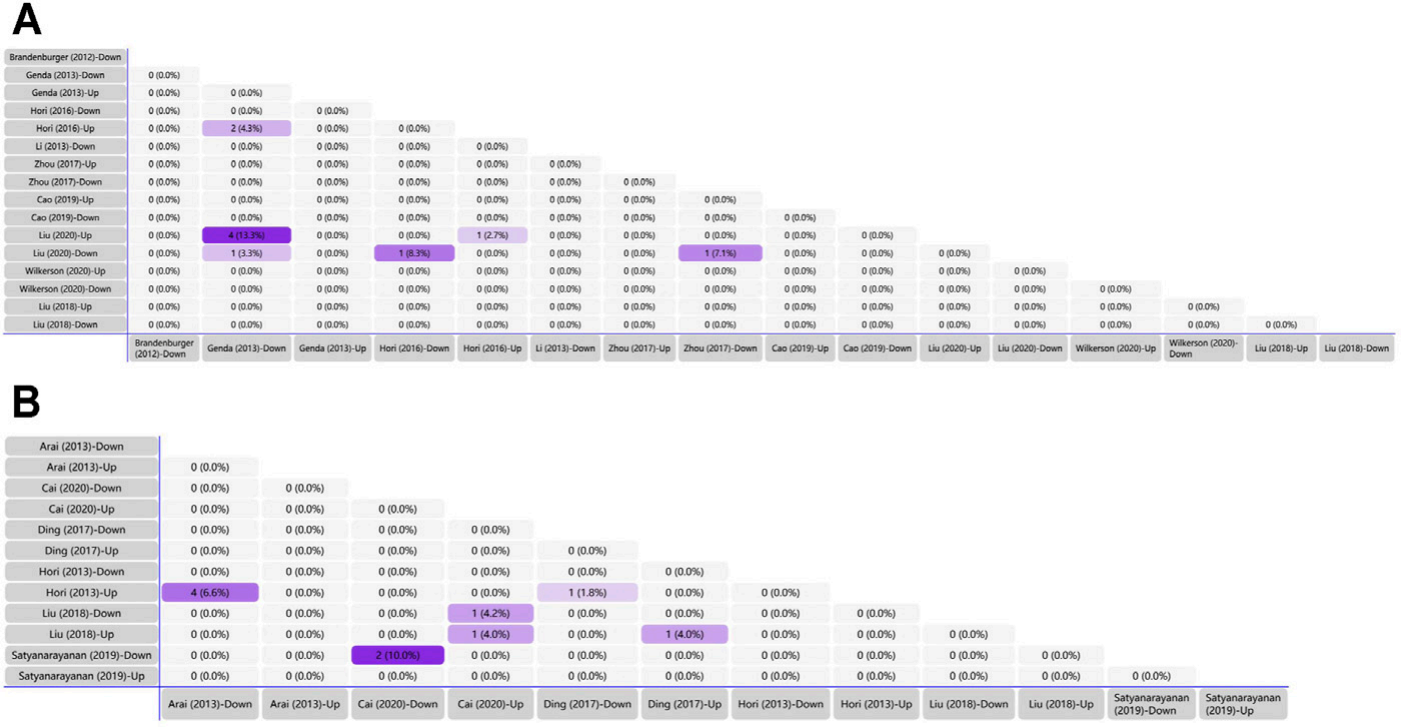
Tabular Venn diagram analysis. **(A)** Matrix table analysis for miRNAs expression profiles in spinal cord of NP surgical models. The number and percentage of co-regulated miRNAs were highlighted. Overlapping miRNAs: 2 (4.3%): miR-21, miR-27b; 4 (13.3%): miR-22, miR-377, miR-7a, miR-21; 1 (3.3%): miR-365; 1 (8.3%): miR-214; 1 (2.7%): miR-21; 1 (7.1%): miR-184. **(B)** Matrix table analysis for miRNAs expression profiles in brain of NP surgical models. The number and percentage of co-regulated miRNAs were highlighted. Overlapping miRNAs: 4 (6.6%): miR-132, miR-151-3p, miR-186, miR-204; 2 (10.0%): miR-200b-3p, miR-182; 1 (4.2%): miR-370-5p; 1 (4.0%) left: miR-873-5p; 1 (4.0%) right: miR-205; 1 (1.8%): miR-539.

**FIGURE 3:**
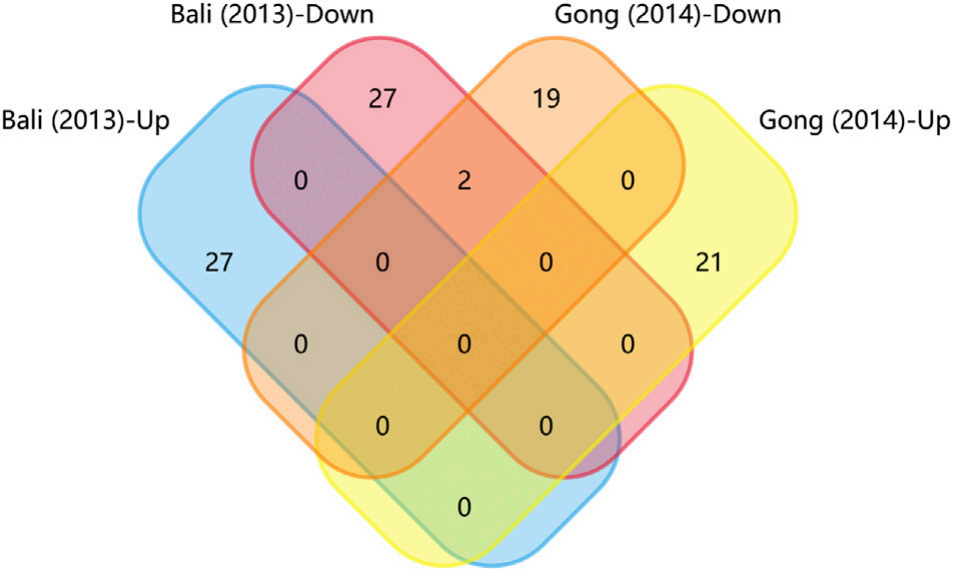
Matrix table analysis for miRNAs expression profiles of disease-induced NP models. The number and percentage of co-regulated miRNAs were highlighted. The two overlapping miRNAs are miR-466i-3p and miR-466g.

**FIGURE 4:**
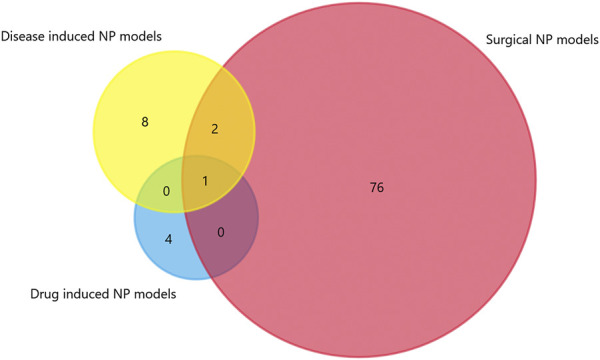
The intersection of experimentally verified miRNAs in three types of rodent NP models. Overlapping miRNAs: 2: miR-34c-5p, miR-145; 1: miR-155.

We generated matrix tables catalogized by DRG, spinal cords and brain tissues using FunRich (http://www.funrich.org), and utilized array data from Supplementary Table S2. It reveals the percentage and quantity of corporately expressed miRNAs in the expression profiling studies. In the matrix table for spinal cords of surgical NP rodents (Figure 2A), miR-365, miR-214 and miR-184 were down-regulated in two or more studies, and miR-21 was up-regulated in two studies. As for brain tissues (Figure 2B), miR-200b-3p and miR-182 were down-regulated in two studies, miR-873-5p was up-regulated, whereas none commonly down-regulated nor up-regulated miRNA in DRGs was found (Supplementary Figure S1). According to array data from Supplementary Table S3, Venn diagram was drawn using FunRich. It shows that miR-466i-3p and miR-466g were commonly down-regulated (Figure 3). Experimentally verified studies were analyzed, and rodent NP models were divided into surgical, disease-induced and drug-induced NP models. The Venn diagram shows that miR-155 was the only miRNA that was verified in all three types of NP models with up-regulated expression; miR-34c-5p was commonly up-regulated and miR-145 was commonly down-regulated in disease-induced and drug-induced NP models (Figure 4).

**FIGURE 5:**
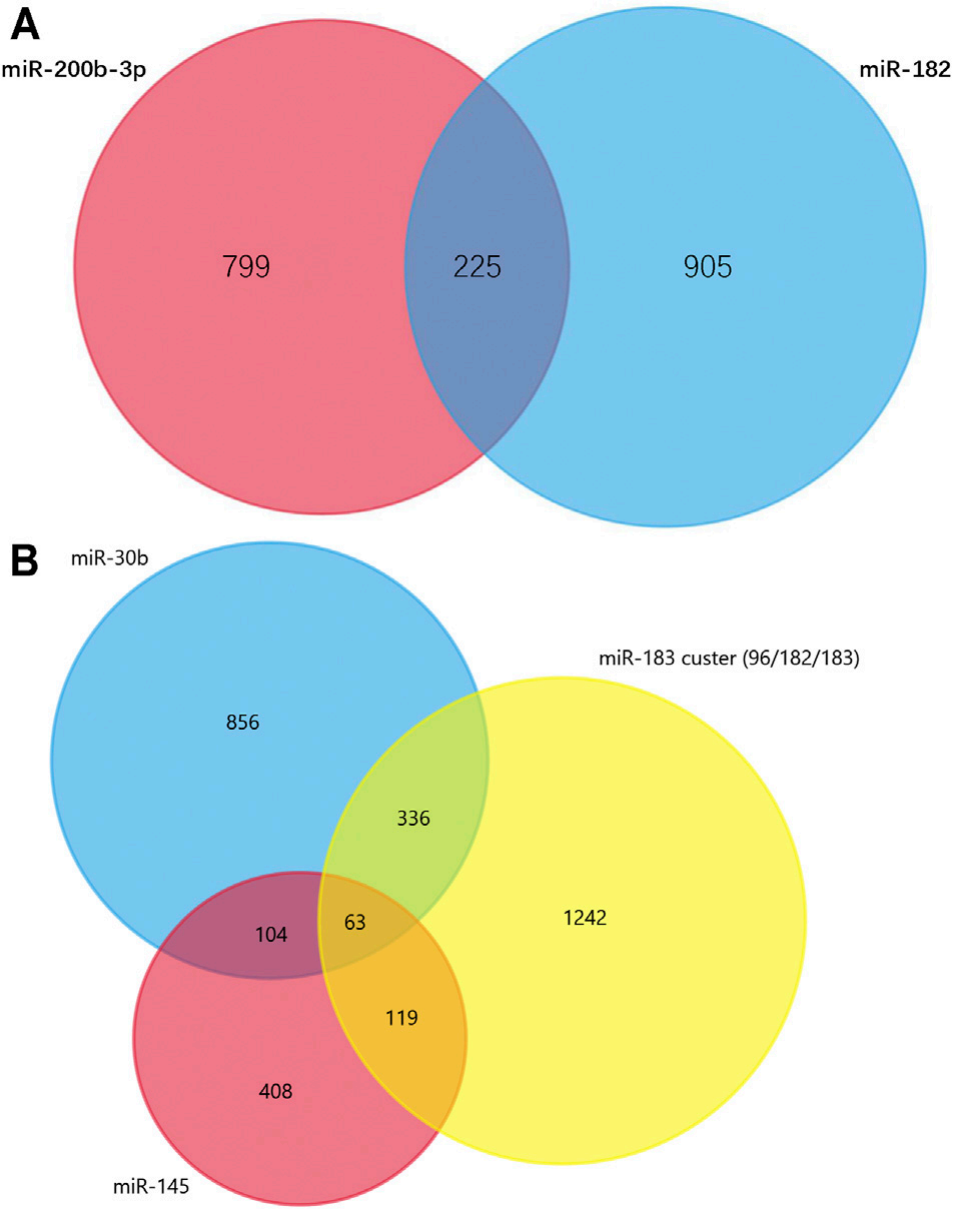
Venn diagram analysis. **(A)** Overlapping target genes of miR-182 and miR-200b in cerebral cortex. These down-regulated genes were showed in Supplementary Table S2 and have been observed in more than one studies. **(B)** Overlapping target genes of miR-96, miR-182, miR-183, miR-145 and miR-30b in dorsal root ganglions. These five down-regulated genes were showed in Supplementary Table S4 and have been observed in two or more experimentally verified studies. miR-96, miR-182, miR-183 formed miR-183 cluster, so their target genes were combined.

TargetScan was used to predict the miRNAs’ target genes and then FunRich was used to draw Venn diagrams. There were 225 overlapping target genes in the two down-regulated miRNAs (miR-200b-3p and miR-182), which detected from the cerebral cortex; but none in the three down-regulated miRNAs (miR-365, miR-214 and miR-184), which detected from spinal cords (Figure 5A and Supplementary Figure S2). Analysis of experimentally verified studies of surgical NP indicated that miR-96, miR-182, miR-183, miR-30b and miR-145 were down-regulated both in spinal cords and DRGs. Furthermore, these five miRNAs were reported in at least two experimental studies. The miR-183 cluster comprised miR-96, miR-182 and miR-183. This cluster shared the same sequence homology, so it is reasonable to merge the target genes of miR-96, miR-182 and miR-183 for Venn diagrams analysis. A total of 63 overlapping target genes were present in the miR-183 cluster, miR-30b and miR-145 (Figure 5B). Moreover, 13 overlapping target genes were found in three repeatedly verified up-regulated miRNAs (miR-21, miR-155 and miR-195) (Supplementary Figure S3).

### PPI Network Analysis

**FIGURE 6:**
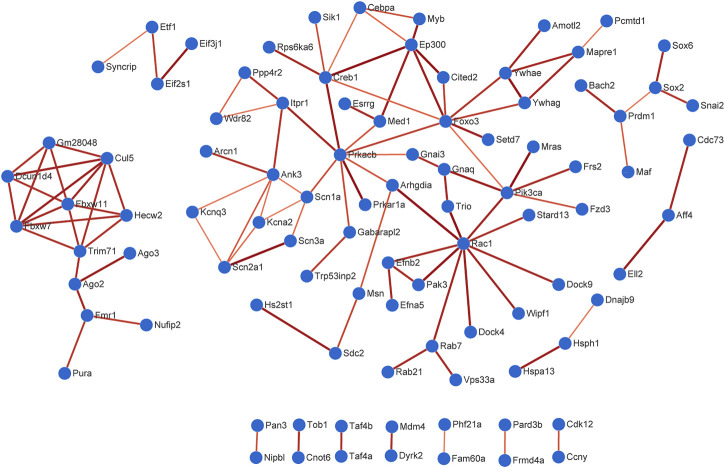
Protein-protein interaction (PPI) analysis. A total of 94 genes exhibited interactions in 225 overlapping target genes of miR-200b-3p and miR-182. Large sizes and dark colors of edges meant high value of combined scores. High confidence score of 0.7 was selected to construction PPI network.

The PPI data of overlapping genes in pain-related nervous tissues were obtained from STRING database, and the interaction networks were constructed using Cytoscape software (v3.7.2). The results of PPI analysis are shown in Figure 6 and Supplementary Figure S4. The results showed 94 genes exhibiting interactions in 225 overlapping target genes of miR-200b-3p and miR-182 (Figure 6). A total of 17 genes showed interactions in 63 overlapping target genes of miR-183 cluster, miR-30b and miR-145 (Supplementary Figure S4). Each edge in the PPI network represented protein-protein associations. Large sizes and dark colors of edges meant high value of combined scores, otherwise, small sizes and bright colors of edges meant low value of combined scores. As shown in Figure 6, muscle and microspikes RAS (Mras) connected with phosphatidylinositol 3-kinase, catalytic, alpha polypeptide (Pik3ca), and cAMP responsive element binding protein 1 (Creb1) connected with E1A Binding Protein P300 (EP300) displayed highest connective score with combined score of 0.997 and 0.996, respectively. In addition, combined score of 0.998 was the highest score in Supplementary Figure S4, which is insulin like Growth factor 1 receptor (Igf1r) connected with insulin receptor substrate 1 (Irs1).

**FIGURE 7:**
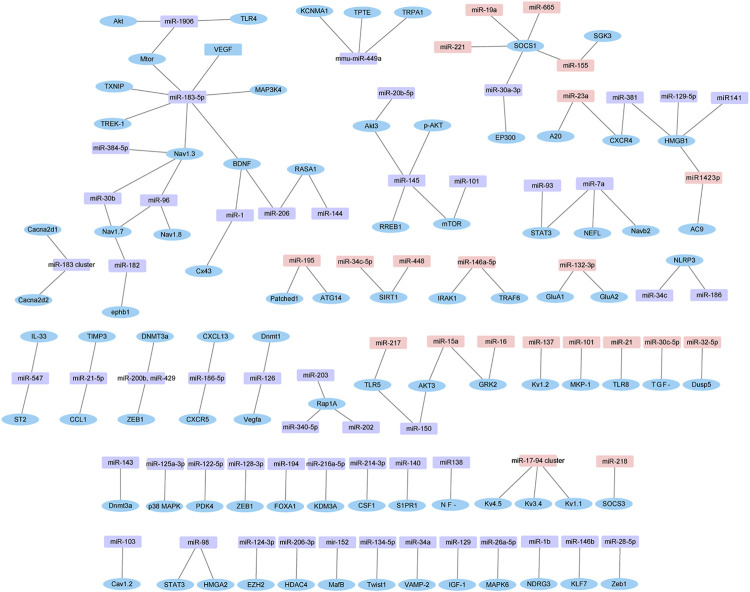
Dysregulated miRNAs-target gene network. The network is based on dysregulated miRNAs and their target genes identified in Supplementary Table S4. Pink in rectangle represent up-regulated miRNAs, purple in rectangle represent down-regulated miRNAs, and blue in ellipse represent target genes.

Furthermore, the experimentally verified miRNAs in Supplementary Table S4 and their corresponding target genes were also mapped (Figure 7). Four down-regulated miRNAs (miR-96, miR-183, miR-384 and miR-30b) directly targeted Nav1.3. Similarly, four up-regulated miRNAs (miR-19a, miR-221, miR-155 and miR-665) and one down-regulated miRNA (miR-30a-3p) directly targeted suppressor of cytokine signaling 1 (SOCS1).

### Functional Enrichment Analysis

**FIGURE 8:**
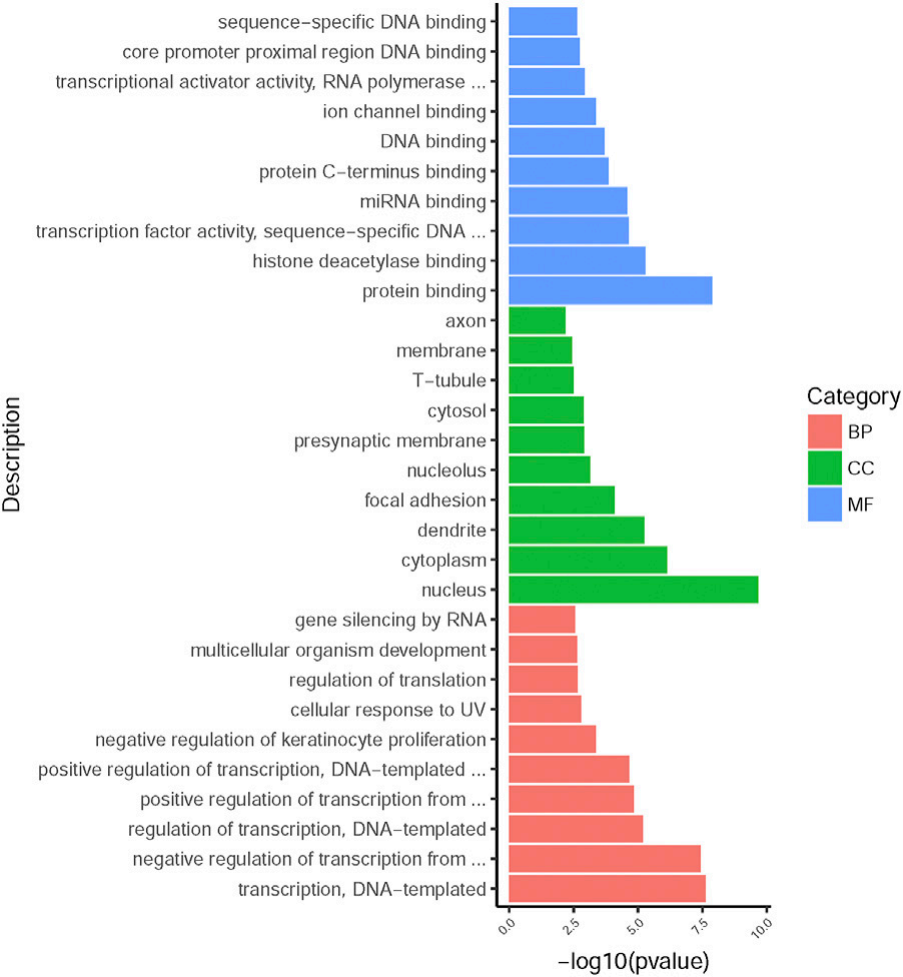
GO annotation enrichment analysis. The top 10 high enrichment score terms in biological process, cellular components and molecular functions. GO: gene ontology; BP: biological process; CC: cellular components; MF: molecular functions.

**FIGURE 9:**
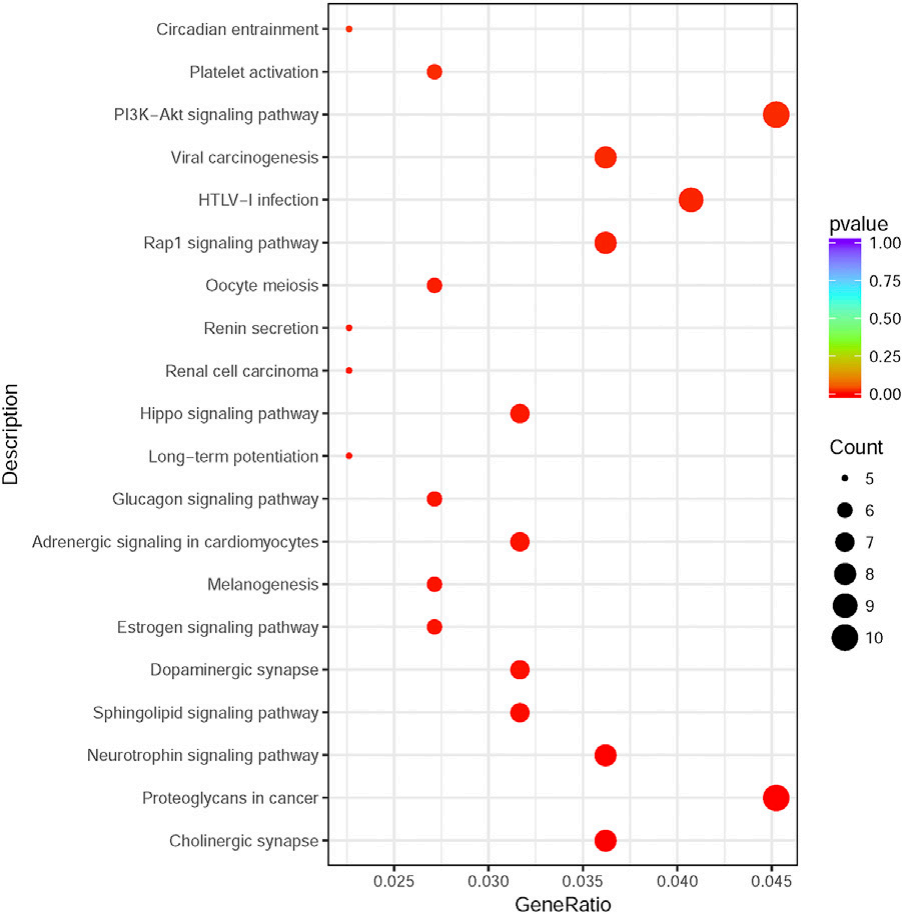
KEGG pathway enrichment analysis. The top 20 enrichment score pathways were showed. KEGG: Kyoto Encyclopedia of Genes and Genomes.

225 overlapping target genes of miR-200b-3p and miR-182 were analyzed. The results of GO analysis consisted of cellular components (e.g., nucleus, GO: 0005634), molecular functions (e.g., protein binding, GO: 0005515), and biological process (e.g., transcription, DNA-templated, GO: 0006351). Overall, 133 GO terms showed significant enrichment (*p* < 0.05), and the top 10 terms with enrichment score in each aspect are presented in Figure 8. A number of enriched terms relevant to the nervous system included dendrite (GO: 0005634), synapse (GO: 0045202), axon (GO: 0030424), nervous system development (GO: 0007399) and neuron projection (GO: 0043005). Furthermore, KEGG analysis showed 26 pathways showed significant enrichment (*p* < 0.05), and the top 20 pathways with enrichment score are presented in Figure 9. Many of the enriched pathways were associated with the nervous system, including cholinergic synapse, neurotrophin signaling pathway, dopaminergic synapse, hippo signaling pathway and glutamatergic synapse.

## Discussion

In this study, miRNAs in nervous tissues of NP rodent models were comprehensively analyzed using 23 miRNA profile articles and 113 miRNA experimental verification articles. Compared with the sham-operated group, a total of 91 miRNAs verified by 113 experimental articles were differentially expressed in the NP group. The potential functional specificity of the differentially expressed miRNAs for pain was determined by GO and KEGG pathway analysis. The results suggest that miRNAs served a vital role in NP development and potentially novel strategies for NP management.

NP pathogenesis is complex and remains poorly understood. Regions of DRGs, SDH and anterior cingulate cortex (ACC) form the somatosensory pathway from primary sensory neurons to afferent nerve to central nervous system (CNS) in NP (Guo et al., 2019). DRGs contain most of the body’s sensory neurons, and transmitted sensory messages from receptors, such as thermoreceptors and nociceptors, are active participants in the signalling process (Pope et al., 2013). SDH is the relay station of nociceptive stimuli to process somatosensory information and is characterized as an unambiguous laminar structure with a quantity of excitatory and inhibitory interneurons (Peirs and Seal, 2016). ACC is a dominant cortical area implicated in diverse neurological processes, such as nociception, cognition and emotion and plays a critical role in emotional/aversive component of pain (Tsuda et al., 2017).

Numerous circuits and molecules were identified as potential biomarkers and regulators in NP. The renowned mediators contain the transient receptor potential (TRP) channels (Lu et al., 2017; Miao et al., 2019; Duan et al., 2020; Zhang and Chen, 2021), voltage-gated sodium channels (Na_V_s) (Chen et al., 2014; Lin et al., 2014; Shao et al., 2016; Su et al., 2017; Cai et al., 2018; Li L et al., 2019; Yang et al., 2019; Yan et al., 2020b; Ye et al., 2020; Sun et al., 2021), and voltage-gated calcium channels (CaVs) (Favereaux et al., 2011; Gandla et al., 2017; Peng et al., 2017). In DRG and SDH, TRP channels act as transducers for selectively activating sensory neurons and conveying diverse kinds of stimuli involving chemical, mechanical, and thermal stimuli. TRP ankyrin 1 (TRPA1) is one of branches of TRP channels contributing to NP. In NP surgical models and drug-induced NP models, TRPA1 is overexpressed and targeted by miR-155 (Miao et al., 2019; Duan et al., 2020), miR-141-5p (Zhang and Chen, 2021), mmu-miR-449a (Lu et al., 2017). Ectopic action potential generation is considered to be one of causes of NP formulation. Na_V_s intrinsically contribute to ectopic activity generation (Bannister et al., 2020) and are typically encoded by different subunits, Na_V_1.1-Na_V_1.9. DRG neurons contain the most varied kind of Na_V_ subtypes. Among them, Na_V_1.3 (Chen et al., 2014; Ye et al., 2020), Na_V_1.6 (Li L et al., 2019), Na_V_1.7 (Sun et al., 2021) and Na_V_1.8 (Yan et al., 2020b) are overexpressed in NP and are targeted by miR-96, miR-183, miR-182 and miR-30b. In addition, Sun and colleagues (Sun et al., 2021) demonstrated that miR-96 knockout mice showed thermal and mechanical allodynia. This allodynia was alleviated by intraperitoneal or intrathecal injection of Na_V_1.7 or Na_V_1.8 blockers. SNI-induced suppressed expression of miR-96 in SDH showed negative correlation to overexpression of Na_V_1.7-Na_V_1.9. This dysregulation promotes the NP process, and can be attenuated by intrathecal injection of corresponding Na_V_s blockers. Thus, alternation in the expression of Na_V_s in nerve injury-induced NP might be efficacious for pain relief. Furthermore, increased expression and function of Ca_V_s also caused NP by increasing transmitter release (Davies et al., 2007). Ca_V_s are grouped into two classes, namely, high-voltage activated type and low-voltage activated type (also known as T-type) (Catterall et al., 2005). Apart from T-type Ca_V_s, Ca_V_s have three types of subunits, namely, the α1 channel-forming subunit, the intracellular *β* subunit and the α2δ auxiliary subunit. The α2δ auxiliary subunit is composed of two disulfide-bonded polypeptides (α2 and δ) (Dolphin, 2018). Furthermore, Ca_V_1.2 that encodes the α1 subunit of Ca_V_s targeted by miR-103 is upregulated in SNL rats (Favereaux et al., 2011); Ca_V_2.3, which targeted by miR-34c-5p, is downregulated in bone metastatic pain mice (Gandla et al., 2017). Both mRNA and protein levels of the α2δ-1 subunit and α2δ-2 subunit of Ca_V_ are upregulated in SNI mice and are correlated with mechanical allodynia (Peng et al., 2017). The miR-183 cluster (miR-183/96/182) controls over 80% of NP-related genes and scales mechanical allodynia by modulating the α2δ-1 subunit and α2δ-2 subunit (Peng et al., 2017). Thus, post-injury alteration in α2δ expression level, bound with upstream miRNAs dysregulations, provides convincing evidence for potential biomarkers and regulators in NP and therapeutic possibilities of Ca_V_s.

Several studies indicated that epigenetic changes, translational modification and post-translational control influence NP development and management. Epigenetic changes are considered to involve histone acetylation and DNA methylation. As such, gene expression is regulated but does not change the coding sequence. DNA methylation likely reduces transcription efficiency, whilst histone acetylation in DNA presents the active transcriptional region (Jenuwein and Allis, 2001). Methyl-CpG-binding protein 2 (MeCP2), a protein with affinity for methylated DNA and repressing transcription from methylated gene promoter, is vital for neuronal proliferation and embryonic development. Manners et al. (Manners et al., 2016) found that SNI caused the redistribution of MeCP2 to methyl-CpG binding domain. Enriched MeCP2 can bind to the miR-126 locus in NP condition and restrain miR-126 transcription. Repressed miR-126 expression contributes to the up-regulation of its two target genes Dnmt1 and Vegfa in SNI mice. DNA methyltransferase 3A (Dnmt3a) was increased in injured DRG. Xu et al. (Xu et al., 2017) indicated that SNL-induced miR-143 downregulation is a negative regulator in Dnmt3a expression in DRG. Furthermore, histone deacetylases (HDACs) were implied in the mechanisms of transcriptional regulation, cell cycle progression, neuron degeneration and regulation of neuronal plasticity. Overexpression of HDAC4 alleviated the effects of miR-206-3p on NP (Wen et al., 2019). Overall, miRNA-regulated DNA methylation and histone acetylation may be a potential target for NP therapeutic management. The interactions between transcriptional factors (TFs) and mitogen-activated protein kinases (MAPKs) are also involved in NP. MAPK6 up-regulation in SDH of CCI rats was deemed to be a direct downstream target gene of miR-26a-5p (Zhu et al., 2018). Suppressed miR-125a-3p contributed to p38 MAPK up-regulation in rat trigeminal ganglions at different time points with prosopalgia (Dong et al., 2014). Inhibition of miR-155 reduces NP during chemotherapeutic bortezomib *via* downstream signals p38-MAPK (Duan et al., 2020). Additionally, in diabetes mellitus rats, miR-133a-3p antagomir administration lightened diabetic NP and down-regulated p38 MAPK (p-p38) phosphorylation (Chang et al., 2020). Hence, pain-related miRNAs and proteins may reveal critical insights into how neurons process incoming stimuli inputs and attempt to create an effective method for low risk of inflammation and NP management.

PPI network analysis may help predicted some target genes to broaden the treatment options for NP in the near future. Mras connected with Pik3ca displayed highest connective score with combined score of 0.997 predicted by PPI network. Mras is a member of the Ras family of small GTPases that impacts mouse embryonic stem cell plasticity and neurogenesis (Mathieu et al., 2013). Pik3ca is usually described as an oncogenic gene and related to PI3K-AKT signaling pathway (Koundouros et al., 2020), which is in line with Figure 9. Another pair with high combined score of 0.996 is Creb1 and EP300. Creb1 encodes a transcription factor. This protein activity is regulated by protein kinase A (Maekawa et al., 2008) and also related to PI3K-AKT signaling pathway (Rodon et al., 2014). Creb1 was recognized as one of most enriched genes in brain and found to regulate a quite number of downstream genes in NP process (Yan et al., 2019). EP300 is identified as a transcriptional co-activator and is important in the processes of cell proliferation and differentiation (Son et al., 2019). Knockdown of miR-30a-3p in L4-6 spinal dorsal toot increased EP300 level and induced NP (Tan et al., 2020). At present, there is no study targeting Mras and Pik3ca in neuropathic pain. Using Pik3ca inhibitors in NP model may be an initial attempt to ameliorate pain in the near future.

Bioinformatics insights into the potential neurobiological mechanisms correlative of NP were investigated in a comprehensive and systematic literature search framework. This framework provided guidance for literature validation of microRNA regulatory networks inferred from real experimental data. An extensive range of miRNAs was obtained to explore the relationship among their downstream genes *via* network and pathway-based analysis. Based on this analysis, it is helpful to form integrative research strategies from NP mechanisms to treatment. Some limitations still exist in this study. Firstly, a few microRNAs revealed contradictory results in NP process, which may be caused by differences in tissue collection timing, modeling methods and detected tissue region. Secondly, the GRADE system to evaluate level of evidence and Funnel plots for publication assessment are lacking. Although the outcomes deduced from bioinformatic analysis, to some extent, may exist heterogeneity from different study designs, network analysis combined pathway analysis can be more robust for possible false positive results.

In summary, we investigated miRNA expression and their target gene roles in NP. Bioinformatics analysis elucidates that several miRNAs (miR-200b-3p, miR-96, miR-182, miR-183, miR-30b, miR-155 and miR-145) and its target genes are involved in known relevant pathways of NP. Targets on voltage-gated sodium channels may be harnessed for pain relief. As knowledge of molecular, genetic and epigenetic mechanisms about pain specificity and neuron plasticity accrues from rodent models, further delineation of signal processing and modulation in neuronal ensembles is key to achieving therapeutic success in future studies.

## Data Availability

The original contributions presented in the study are included in the article/Supplementary Material, further inquiries can be directed to the corresponding authors.
