# Supplementary material for: microRNA-Based Network and Pathway Analysis for Neuropathic Pain in Rodent Models
Source: Front Mol Biosci. 2022 Jan 13;8:780730. doi: 10.3389/fmolb.2021.780730 (PMC8794747; doi:10.3389/fmolb.2021.780730)
Supplement: Supplementary file 1 [file Table1.DOCX]

Jianguoyun download link for Source data:

<https://www.jianguoyun.com/p/DVCAkiUQoNbcCRjRiIoE>
