# Supplementary material for: microRNA-Based Network and Pathway Analysis for Neuropathic Pain in Rodent Models
Source: Front Mol Biosci. 2022 Jan 13;8:780730. doi: 10.3389/fmolb.2021.780730 (PMC8794747; doi:10.3389/fmolb.2021.780730)
Supplement: Supplementary file 2 [file Table2.DOCX]

Supplementary Material

# Supplementary Information

**Appendix S1. Search strategies for all databases**

1. Search Strategy for Pubmed

#1 MicroRNAs[Mesh Terms] OR "microRNA*"[Text Word] OR "mir*"[Text Word] OR "micro RNA"[Text Word] OR "micro RNAs"[Text Word] OR "micro-RNA"[Text Word] OR "micro-RNAs"[Text Word]

#2 Sciatica[Mesh Terms] OR "sciatica*"[Text Word] OR "ischial*"[Text Word] OR "sciatic neuropathy"[Text Word] OR "sciatic pain"[Text Word] OR "sciatic nerve palsy"[Text Word] OR "sciatic nerve diseases"[Text Word] OR "sciatic neuritis"[Text Word] OR "chronic constriction injury"[Text Word] OR "CCI"[Text Word] OR "partial sciatic nerve injury"[Text Word] OR "PNI"[Text Word] OR "spinal nerve ligation"[Text Word] OR "SNL"[Text Word] OR "chronic compression"[Text Word] OR "CCD"[Text Word] OR "spared nerve injury"[Text Word] OR "SNI"[Text Word]

#3 Neuralgia[Mesh] OR Neuralgia*[Title/Abstract] OR Neurodynia*[Title/Abstract] OR "Neuropathic pain*"[Title/Abstract] OR “Nerve pain*”[Title/Abstract] OR sciatica[Title/Abstract]

#4 #2 OR #3

#5 #1 AND #4

2. Search Strategy for Web of Science

#1 TS=("MicroRNA*" OR "mir*" OR "micro RNAs" OR "micro RNA" OR "micro-RNAs" OR "micro-RNA")

#2 TS=("sciatica*" OR "ischial*" OR "sciatic neuropathy" OR "sciatic pain" OR "sciatic nerve palsy" OR "sciatic nerve diseases" OR "sciatic neuritis" OR "chronic constriction injury" OR "CCI" OR "partial sciatic nerve injury" OR "PNI" OR "spinal nerve ligation" OR "SNL" OR "chronic compression dorsal root ganglion" OR "CCD" OR "spared nerve injury" OR "SNI")

#3 TS=( Neuralgia$ OR Neurodynia$ OR "Neuropathic pain$")

#4 #2 OR #3

#5 #1 AND #4

Timespan=All years. Databases=SCI-EXPANDED, SSCI.

3. Search Strategy for EMBASE:

#1 'MicroRNA'/exp OR 'mir*':ab,ti OR 'microRNAs':ab,ti OR 'microRNA':ab,ti OR 'micro RNAs':ab,ti OR 'micro RNA':ab,ti OR 'micro-RNAs':ab,ti OR 'micro-RNA':ab,ti

#2 'sciatica'/exp OR 'sciatica*':ab,ti OR 'ischial*':ab,ti OR 'sciatic neuropathy':ab,ti OR 'sciatic pain':ab,ti OR 'sciatic nerve palsy':ab,ti OR 'sciatic nerve diseases':ab,ti OR 'sciatic neuritis':ab,ti OR 'chronic constriction injury':ab,ti OR 'CCI':ab,ti OR 'partial sciatic nerve injury':ab,ti OR 'PNI':ab,ti OR 'spinal nerve ligation':ab,ti OR 'SNL':ab,ti OR 'chronic compression dorsal root ganglion':ab,ti OR 'CCD':ab,ti OR 'spared nerve injury':ab,ti OR 'SNI':ab,ti

#3 'neuralgia'/exp OR neuralgia OR neuralgia$:ab,ti OR neurodynia$:ab,ti OR "neuropathic pain$":ab,ti OR "nerve pain$":ab,ti

#4 #2 OR #3

#5 #1 AND #4

4. Search Strategy for CINAHL Complete(EBSCO)

S1 TX("MicroRNA*" OR "mir*" OR "micro RNAs" OR "micro RNA" OR "micro-RNAs" OR "micro-RNA")

S2 TX("sciatica*" OR "ischial*" OR "sciatic neuropathy" OR "sciatic pain" OR "sciatic nerve palsy" OR "sciatic nerve diseases" OR "sciatic neuritis" OR "chronic constriction injury" OR "CCI" OR "partial sciatic nerve injury" OR "PNI" OR "spinal nerve ligation" OR "SNL" OR "chronic compression dorsal root ganglion" OR "CCD" OR "spared nerve injury" OR "SNI")

S3 TX("Neuralgia" OR "Neuralgia*" OR "Neurodynia*" OR "Neuropathic pain*" OR “Nerve pain*”)

S4 S2 OR S3

S5 S4 AND S5

# Supplementary Figures and Tables

## Supplementary Table

Table S1. List of excluded studies

| **No.** | **Reason** | **Title** |
| --- | --- | --- |
| 1 | non-miRNA analysis | Differential expression of microRNAs in mouse pain models |
| 2 | non-miRNA analysis | MicroRNA-143 expression in dorsal root ganglion neurons |
| 3 | withdrawn | MiR-216b-5p attenuates chronic constriction injury-induced neuropathic pain in female rats by targeting MAL2 and inactivating Wnt/β-catenin signaling pathway |
| 4 | visceral pain | MicroRNA-330 Directs Downregulation of the GABA(B)R2 in the Pathogenesis of Pancreatic Cancer Pain |
| 5 | spinal cord injury | Down-regulation of miRNA-128 contributes to neuropathic pain following spinal cord injury via activation of P38 |
| 6 | spinal cord injury | PEITC promotes neurite growth in primary sensory neurons via the miR-17-5p/STAT3/GAP-43 axis |
| 7 | spinal cord injury | miR-30b Promotes spinal cord sensory function recovery via the Sema3A/NRP-1/PlexinA1/RhoA/ROCK Pathway |
| 8 | spinal cord injury | MicroRNA-331-3P attenuates neuropathic pain following spinal cord injury via targeting RAP1A |
| 9 | spinal cord injury | Knockdown of miR-130a-3p alleviates spinal cord injury induced neuropathic pain by activating IGF-1/IGF-1R pathway |
| 10 | review | microRNAs in nociceptive circuits as predictors of future clinical applications |
| 11 | review | Noncoding RNAs: key molecules in understanding and treating pain |
| 12 | review | On the prospect of clinical utilization of microRNAs as biomarkers or treatment of chronic pain |
| 13 | review | MicroRNA Mediated Regulation of Schwann Cell Migration and Proliferation in Peripheral Nerve Injury |
| 14 | review | The role of microRNAs in the healing of diabetic ulcers |
| 15 | review | Tissue Specific Reference Genes for MicroRNA Expression Analysis in a Mouse Model of Peripheral Nerve Injury |
| 16 | review | Identification of candidate genes and miRNAs associated with neuropathic pain induced by spared nerve injury |
| 17 | review | Analysis of Crucial Genes and Pathways Associated with Spared Nerve Injury-Induced Neuropathic Pain |
| 18 | Retracted article | miR23b ameliorates neuropathic pain in spinal cord by silencing NADPH oxidase 4 |
| 19 | non-miRNA analysis | Identification and functional analysis of novel micro-RNAs in rat dorsal root ganglia after sciatic nerve resection |
| 20 | non-miRNA analysis | Sources of individual variability: MiRNAs that predispose to neuropathic pain identified using genome-wide sequencing |
| 21 | non-miRNA analysis | MicroRNAs downregulated in neuropathic pain regulate MeCP2 and BDNF related to pain sensitivity |
| 22 | non-miRNA analysis | High-resolution transcriptome analysis reveals neuropathic pain gene-expression signatures in spinal microglia after nerve injury |
| 23 | non-miRNA analysis | MicroRNA-338 and microRNA-21 co-transfection for the treatment of rat sciatic nerve injury |
| 24 | non-miRNA analysis | Chronic constriction injury of sciatic nerve changes circular RNA expression in rat spinal dorsal horn |
| 25 | non-miRNA analysis | Identification of Key Gene Modules of Neuropathic Pain by Co-Expression Analysis |
| 26 | non-miRNA analysis | Bioinformatics Analysis of Genes and Mechanisms in Postherpetic Neuralgia |
| 27 | non-miRNA analysis | Identification of key candidate genes in neuropathic pain by integrated bioinformatic analysis |
| 28 | non-miRNA analysis | Suppression of miR-155 attenuates neuropathic pain by inducing an M1 to M2 switch in microglia |
| 29 | non-miRNA analysis | TRPV1, Targeted by miR-338-3p, Induces Neuropathic Pain by Interacting with NECAB2 |
| 30 | morphine-induced pain model | MicroRNA-124 and microRNA-146a both attenuate persistent neuropathic pain induced by morphine in male rats |
| 31 | cell experiment | MicroRNA-9 regulates mammalianaxon regeneration in peripheral nerve injury |
| 32 | meta-analysis | microRNA-9 and -29a regulate the progression of diabetic peripheral neuropathy via ISL1-mediated sonic hedgehog signaling pathway |
| 33 | lncRNA research | SNHG16 aggravates chronic constriction injury-induced neuropathic pain in rats via binding with miR-124-3p and miR-141-3p to upregulate JAG1 |
| 34 | lncRNA research | XIST/miR-544 axis induces neuropathic pain by activating STAT3 in a rat model |
| 35 | lncRNA research | NEAT1 contributes to neuropathic pain development through targeting miR-381/HMGB1 axis in CCI rat models |
| 36 | lncRNA research | XIST accelerates neuropathic pain progression through regulation of miR-150 and ZEB1 in CCI rat models |
| 37 | lingual nerve sample | Correlation of miRNA expression with intensity of neuropathic pain in man |
| 38 | investigate the role of upstream genes | MeCP2 plays an analgesic role in pain transmission through regulating CREB/miR-132 pathway |
| 39 | investigate the role of upstream genes | Activating Sirt1 by resveratrol suppresses Nav1.7 expression in DRG through miR-182 and alleviates neuropathic pain in rats |
| 40 | investigate the role of upstream genes | A critical role for miR-135a-5p-mediated regulation of SLC24A2 in neuropathic pain |
| 41 | Hindpaw incision model | miR-203 Regulates Nociceptive Sensitization after Incision by Controlling Phospholipase A2 Activating Protein Expression |
| 42 | drug intervention | Effects of microRNA-223 on morphine analgesic tolerance by targeting NLRP3 in a rat model of neuropathic pain |
| 43 | drug intervention | Intrathecal Injection of miR-133b-3p or miR-143-3p Prevents the Development of Persistent Cold and Mechanical Allodynia Following a Peripheral Nerve Injury in Rats |
| 44 | drug intervention | Propofol ameliorated diabetic peripheral neuropathic pain via modulating miR-150/EPHB2 axis |
| 45 | drug intervention | Granulocyte Colony Stimulating Factor (GCSF) Can Attenuate Neuropathic Pain by Suppressing Monocyte Chemoattractant Protein-1 (MCP-1) Expression, through Upregulating the Early MicroRNA-122 Expression in the Dorsal Root Ganglia |
| 46 | drug intervention | Dexmedetomidine alleviates diabetic neuropathic pain by inhibiting microglial activation via regulation of miR-618/P2Y12 pathway |
| 47 | drug intervention | Dexmedetomidine alleviates microglial activation of neuropathic pain by modulating miR-23a /PDE10A axis in streptozotocin-induced diabetic mice |
| 48 | drug intervention | Low concentration of Bupivacaine ameliorates painful diabetic neuropathy by mediating miR-23a/PDE4B axis in microglia |
| 49 | conference abstract | Changes of miRNA in the amygdala under the neuropathic pain |
| 50 | conference abstract | Decreased expressions of microRNAs in the injured DRG neurons of neuropathic pain rats |
| 51 | conference abstract | miR-7a alleviates the maintenance of neuropathic pain by regulating the voltage-gated sodium channel beta 2 subunit |
| 52 | conference abstract | Multiple profiling of changes in miRNA expression under the neuropathic pain |
| 53 | conference abstract | Micro-RNA 200b/429 in the nucleus accumbens plays a key role in the neuropathic pain |
| 54 | conference abstract | miR-17-92 cluster upregulation in the dorsal root ganglion in neuropathic pain |
| 55 | conference abstract | Involvement of miR-15b in oxaliplatin-induced neuropathic pain |
| 56 | conference abstract | miR-17-92 cluster modulates neuropathic pain and neurite outgrowth |
| 57 | conference abstract | Extracellular miRNA causes neuropathic pain via spinal TLR7 in peripheral nerve injury |
| 58 | conference abstract | Bioinformatic Prediction of Neuropathic Pain Signaling Pathways in Rheumatoid Arthritis after high throughput miRNA analysis |
| 59 | comment | Key Gene Modules of Neuropathic Pain Identified Through Bioinformatics Analyses |
| 60 | visceral pain | Decreased miR-325-5p Contributes to Visceral Hypersensitivity Through Post-transcriptional Upregulation of CCL2 in Rat Dorsal Root Ganglia |
| 61 | CFA-injected chronic inflammatory Pain | MicroRNA-16 Alleviates Inflammatory Pain by Targeting Ras-Related Protein 23 (RAB23) and Inhibiting p38 MAPK Activation |
| 62 | CFA-injected chronic inflammatory Pain | Regulation of μ-opioid type 1 receptors by microRNA134 in dorsal root ganglion neurons following peripheral inflammation |
| 63 | CFA-injected chronic inflammatory Pain | Integrated analysis of microRNA and mRNA expression profiles in the rat spinal cord under inflammatory pain conditions |
| 64 | CFA-injected chronic inflammatory Pain | Spinal miR-34a regulates inflammatory pain by targeting SIRT1 in complete Freund's adjuvant mice |
| 65 | blood sample | miR-124a and miR-155 enhance differentiation of regulatory T cells in patients with neuropathic pain |
| 66 | blood sample | Circulating microRNA Signatures in Rodent Models of Pain |
| 67 | blood sample | Differences in the miRNA signatures of chronic musculoskeletal pain patients from neuropathic or nociceptive origins |
| 68 | blood sample | MIR-101 relates to chronic peripheral neuropathic pain through targeting KPNB1 and regulating NF-κb signaling |

Table S2. Expression profiles of miRNA in NP surgical models

| **Article, Year** | **Country** | **Expression** | **miRNAs** | **Experimental models** | **Method** |
| --- | --- | --- | --- | --- | --- |
| Rau et al., 2010 ([Rau et al., 2010](#_ENREF_17)) | China (Taiwan) | up | miR-21 | L4–L6 DRGs from sciatic nerve transection rats | microarray |
|  |  | down | miR-144 |  |  |
| Schack et al., 2011 ([von Schack et al., 2011](#_ENREF_19)) | USA | up | hsa-miR-486, hsa-miR-30a-3p, hsa-miR-206, hsa-miR-133b | L4 DRG from SNL rats | TLDA |
|  |  | down | hsa-miR-221, hsa-miR-34a, hsa-let-7e, hsa-miR-132, hsa-miR-378, hsa-miR-34c, hsa-miR-409-5p, hsa-miR-135a, hsa-miR-18a, hsa-miR-17-3p, hsa-let-7a, hsa-miR-21, hsa-miR-10b, hsa-let-7d, hsa-miR-93, hsa-miR-20a, hsa-miR-497, hsa-let-7b, hsa-miR-10a, hsa-let-7c, hsa-miR-142-3p, hsa-miR-27b, hsa-let-7g, hsa-miR-301, hsa-miR-324-5p, hsa-miR-133a, hsa-miR-20b, hsa-miR-125b, hsa-miR-27a, hsa-miR-148b, hsa-miR-369-5p, hsa-miR-92, hsa-miR-181c, hsa-miR-100, hsa-miR-148a, hsa-miR-383, hsa-miR-9, hsa-miR-127, hsa-miR-26b, hsa-miR-30a-5p, hsa-miR-142-5p, hsa-miR-30d, hsa-miR-190, hsa-miR-19a, hsa-miR-23b, hsa-miR-339, hsa-miR-137, hsa-miR-181b, hsa-miR-19b, hsa-miR-126, hsa-miR-218, hsa-miR-181d, hsa-miR-335, hsa-miR-103, hsa-miR-26a, hsa-miR-299-5p, hsa-miR-572, hsa-miR-659, hsa-miR-338 |  |  |
| Brandenburger et al., 2012 ([Brandenburger et al., 2012](#_ENREF_2)) | Germany | down | mmu-miR-30b, mmu-miR-100, mmu-miR-10a, mmu-miR-99a, mmu-miR-582-3p, mmu-miR-720 | L4–L6 spinal cord from CCI rats | Microarray, qPCR |
| Arai et al., 2013 ([Arai et al., 2013](#_ENREF_1)) | Japan | up | hsa-miR-22, hsa-miR-338 | hippocampus from CCI rats | TLDA |
|  |  | down | mmu-miR-124, mmu-miR-132, mmu-miR-151-3p, mmu-miR-186, mmu-miR-187, mmu-miR-204, mmu-miR-210, mmu-miR-25, mmu-miR-27a, mmu-miR-30e, mmu-miR-34c, mmu-miR-448, mmu-miR-449a, mmu-miR-488, mmu-miR-668, mmu-miR-92a, mmu-miR-98, rno-miR-1 |  |  |
| Genda et al., 2013 ([Genda et al., 2013](#_ENREF_10)) | Japan | up | mmu-miR-539, rno-miR-381, mmu-miR-323-3p | L4-L5 SDH from CCI rats | TLDA |
|  |  | down | hsa-miR-22, mmu-miR-496, mmu-miR-151-3p, mmu-miR-24-2, mmu-miR-324-5p, rno-miR-345-3p, mmu-miR-127, mmu-miR-125b-5p, mmu-miR-221, mmu-miR-296-5p, rno-miR-377, mmu-miR-365, mmu-miR-598, mmu-miR-7a, mmu-miR-101b, mmu-miR-29b, rno-miR-336, hsa-miR-493-3p, mmu-miR-322, mmu-miR-21, mmu-miR-27b, rno-miR-632 |  |  |
| Hori et al., 2013 ([Hori et al., 2013](#_ENREF_12)) | Japan | up | hsa-miR-324-3p, mmu-miR-125a-5p, mmu-miR-132, mmu-miR-151-3p, mmu-miR-17, mmu-miR-191, mmu-miR-222, mmu-miR-31, mmu-miR-320, mmu-miR-434-3p, mmu-miR-539, rno-miR-345-3p, hsa-miR-30a-3p, hsa-miR-30e-3p, mmu-miR-126-3p, mmu-miR-133a, mmu-miR-140, mmu-miR-150, mmu-miR-212, mmu-miR-30a, mmu-miR-323-3p, mmu-miR-331-3p, mmu-miR-383, mmu-miR-431, mmu-miR-487b, mmu-miR-770-5p, mmu-miR-872, rno-miR-125b, rno-miR-146b, rno-miR-339-3p, rno-miR-409-3p, rno-miR-504, rno-miR-664, rno-miR-7a, hsa-miR-28-3p, hsa-miR-423-3p, mmu-miR-128a, mmu-miR-134, mmu-miR-138, mmu-miR-186, mmu miR-193b, mmu-miR-204, mmu-miR-23b, mmu-miR-24, mmu-miR-380-5p, mmu-miR-384-5p, rno-miR-351 | hippocampus from CCI rats | TLDA |
|  |  | down | mmu-miR-181c, mmu-miR-29c, rno-miR-381, rno-miR-632, mmu-miR-30d, mmu-miR-325, mmu-miR-376b |  |  |
| Li et al., 2013 ([Li et al., 2013](#_ENREF_13)) | China | up | rno-miR-341 | L4-L6 DRGs from bCCI rats | Microarray |
|  |  | down | rno-miR-203, rno-miR-181a-1, rno-miR-541 | L2–L4 SDH from bCCI rats |  |
| Dong et al., 2014 ([Dong et al., 2014](#_ENREF_9)) | China | up | miR-24-2, miR-26a, miR-183 | TG from CFA-injected trigeminal neuralgia rats | Microarray, qRT-PCR |
|  |  | down | miR-23a, miR-92a, miR-125a-3p, miR-299 |  |  |
| Hori et al., 2016 ([Hori et al., 2016](#_ENREF_11)) | Japan | up | mmu-miR-431, mmu-miR-511-3p, mmu-miR-204, mmu-miR-21, mmu-miR-92b, mmu-miR-409-3p, mmu-miR-154, mmu-miR-146b, mmu-miR-449a, mmu-miR-667, mmu-miR-434-3p, mmu-miR-5111, mmu-miR-700, mmu-miR-3473c, mmu-miR-361, mmu-miR-3096b-3p, mmu-miR-27b, mmu-miR-18a, mmu-miR-30c-1, mmu-miR-376c, mmu-miR-192, mmu-miR-380-3p, mmu-miR-130b, mmu-miR-380-5p, mmu-miR-223, mmu-miR-466j | L2–L4 SDH from bCCI rats | TLDA |
|  |  | down | mmu-miR-1981, mmu-miR-214, mmu-miR-505-5p, mmu-miR-133a |  |  |
| Chang et al., 2017 ([Chang et al., 2017](#_ENREF_5)) | China | up | miR-146b, miR-21, miR-21-3p, miR-221, miR-222, miR-31, miR-339-3p, miR-344b-1-3p, miR-3566, miR-3574, miR-3596d, miR-466b-1, miR-466b-2, miR-466c | L5 DRG from SNL rats | Microarray |
|  |  | down | miR-122, miR-125b-3p, miR-214, miR-297, miR-32-3p, miR-351-3p, miR-3560, miR-3584-5p, miR-3588, miR-363-5p, miR-466b, miR-466c, miR-466d, miR-664-1-5p, miR-664-2-5p, miR665, miR-668, miR-672, miR-92a-2-5p, miR-99b-3p |  |  |
| Ding er al., 2017 ([Ding et al., 2017](#_ENREF_8)) | China | up | miR-493, miR-205, miR-203, miR-194, miR-380, miR-21, miR-341, miR-221, miR-499 | ACC form CCI rats | Microarray, qRT-PCR |
|  |  | down | miR-192, miR-144, miR-500, miR-340-5p, miR-327, miR-296, miR-539, miR-505, miR-214, miR-129, miR-223 |  |  |
| Lu et al., 2017 ([Lu et al., 2017](#_ENREF_16)) | China | up | mmu-miR-449a, mmu-miR-185 | DRG from SNI mice | Microarray, RT-qPCR |
|  |  | down | NR |  |  |
| Zhou et al., 2017 ([Zhou et al., 2017](#_ENREF_21)) | China | up | rno-miR-101a-3p, rno-miR-29a-3p, rno-miR-490-3p, rno-miR-3556a, rno-miR-29c-3p | L4-L5 SDH form SNI rats | Microarray |
|  |  | down | rno-miR-184, rno-miR-344b-1-3p, rno-miR-666-5p, rno-miR-92a-3p, rno-miR-10a-5p, rno-miR-3556b |  |  |
| Liu et al., 2018 ([Liu et al., 2018](#_ENREF_15)) | China | up | miR-1193-3p, miR-410-5p, miR-340-5p | SDH from CBPA rats | Microarray |
|  |  | down | miR-3573-5p, miR-3074 |  |  |
|  |  | up | miR-205, miR-25-5p, miR-702-3p, miR-501-3p, let-7f-5p, miR-381-5p, miR-30c-1-3p, miR-671, miR-184 | AC from CBPA rats |  |
|  |  | down | miR-208a-3p, miR-6216, miR-3580-3p |  |  |
|  |  | up | miR-3570, miR-3588, miR-664-3p, miR-488-3p, miR-30c-1-3p, miR-106b-3p, miR-93-3p, miR-28-5p, miR-873-5p | thalamus from CBPA rats |  |
|  |  | down | miR-34b-5p, miR-181d-3p, miR-484, miR-370-5p, miR-9b-5p, miR-1912-3p, miR-759, miR-463-5p, miR-193-3p, miR-802-5p, miR-218a-5p, miR-31b, miR-3593-5p |  |  |
| Cao et al., 2019 ([Cao et al., 2019](#_ENREF_4)) | China | up | rno-miR-1b, rno-miR-98-5p, rno-miR-31a-3p, rno-miR-376b-5p, rno-miR-1-3p, rno-miR-214-3p | L3-L5 SDH from CCI rats | TLDA |
|  |  | down | rno-miR-329-5p, rno-miR-675-5p, rno-miR-342-5p, rno-miR-203a-3p, rno-let-7d-5p, rno-miR-542-5p, rno-miR-672-5p |  |  |
| Dai et al., 2019 ([Dai et al., 2019](#_ENREF_7)) | China | up | NR | L3-L6 DRGs from SNI rats | Microarray, RT-qPCR |
|  |  | down | rno-miR-6215, rno-miR-1224, rno-miR-1249, rno-miR-488-3p |  |  |
| Satyanarayanan et al., 2019 ([Satyanarayanan et al., 2019](#_ENREF_18)) | China  (Macao) | up | rno-miR-122-5p, rno-miR-219a-5p, rno-miR-21-3p, rno-miR-33-5p, rno-miR-6216. rno-miR-881-3p | PFC form SNI rats | NGS |
|  |  | down | rno-miR-130b-3p, rno-miR-200b-3p, rno-miR-183-5p, rno-miR-182, rno-miR-200a-3p, rno-miR-429, rno-miR-10a-5p |  |  |
| Cai et al., 2020 ([Cai et al., 2020](#_ENREF_3)) | China | up | rno-miR-873-5p, rno-miR-370-5p, rno-mir-30e, rno-miR-3594-5p, rno-miR-128-1-5p, rno-miR-1-3p, rno-miR-6324, rno-miR-214-3p, rno-miR-350 | prelimbic cortex from SNI rats | Microarray |
|  |  | down | rno-miR-24-1-5p, rno-miR-322-5p, rno-miR-872-3p, rno-miR-380-3p, rno-miR-200b-3p, rno-miR-17-1-3p, rno-miR-191a-3p, rno-miR-667-5p, rno-miR-758-3p, rno-miR-133a-3p, rno-miR-193-5p, rno-miR-877, rno-mir-378b, rno-miR-182, rno-miR-328a-5p |  |  |
| Liu et al., 2020 ([Liu et al., 2020](#_ENREF_14)) | China | up | miR-7a, miR-21, miR-377, miR-218a, miR-493, miR-137, miR-9a, miR-375, miR-1839, miR-448, miR-203a, miR-22 | L4-L6 SDH from SNL rats | Microarray |
|  |  | down | miR-344a-2, miR-211, miR-483, miR-145, miR-378a, miR-365, miR-214, mmu-let-7e, miR-184 |  |  |
| Wilkerson et al., 2020 ([Wilkerson et al., 2020](#_ENREF_20)) | USA | up | miR-299a-5p | L4-L6 SDH from CCI mice | Microarray, qRT-PCR |
|  |  | down | miR-880-5p, miR-466d-5p, miR-469-5p |  |  |
|  |  | up | miR-142-5p | sciatic nerve from CCI mice |  |
|  |  | down | miR-138-5p, miR-138-3p, miR-142-5p, miR-676-3p, miR-183-5P, miR-182-5p, miR-96-5p |  |  |
| Chen et al., 2021 ([Chen et al., 2021](#_ENREF_6)) | China | up | NR | DRG form SNI mice | DESeq2, qRT-PCR |
|  |  | down | miR-125a-5p, miR-125b-5p, miR-351-5p |  |  |

Abbreviations: AC: anterior cingulate; ACC: anterior cingulate cortex; bCCI: bilateral chronic constriction sciatic nerve injury; CBPA: complete brachial plexus avulsion; CCI: chronic constriction sciatic nerve injury; CFA: Freund’s adjuvant; DRG: dorsal root ganglion; NGS microRNA next-generation sequencing; NP: neuropathic pain; PFC: prefrontal cortex; SDH: spinal dorsal horn; SNI: spared nerve injury; SNL: spinal nerve ligation; TGs: trigeminal ganglions; TLDA: Taqman Low Density Array.

References

Arai, M., Genda, Y., Ishikawa, M., Shunsuke, T., Okabe, T., and Sakamoto, A. (2013). The miRNA and mRNA Changes in Rat Hippocampi after Chronic Constriction Injury. *Pain Medicine* 14**,** 720-729.

Brandenburger, T., Castoldi, M., Brendel, M., Grievink, H., Schlösser, L., Werdehausen, R., Bauer, I., and Hermanns, H. (2012). Expression of spinal cord microRNAs in a rat model of chronic neuropathic pain. *Neuroscience Letters* 506**,** 281-286.

Cai, G.H., Zhu, Y.Y., Zhao, Y., Chen, J., Guo, C.H., Wu, F.F., Huang, J., and Wu, S.X. (2020). Network Analysis of miRNA and mRNA Changes in the Prelimbic Cortex of Rats With Chronic Neuropathic Pain: Pointing to Inflammation. *Frontiers In Genetics* 11.

Cao, S., Yuan, J., Zhang, D., Wen, S., Wang, J., Li, Y., and Deng, W. (2019). Transcriptome changes in dorsal spinal cord of rats with neuropathic pain. *Journal of Pain Research* 12**,** 3013-3023.

Chang, H.L., Wang, H.C., Chunag, Y.T., Chou, C.W., Lin, I.L., Lai, C.S., Chang, L.L., and Cheng, K.I. (2017). miRNA Expression Change in Dorsal Root Ganglia After Peripheral Nerve Injury. *Journal of Molecular Neuroscience* 61**,** 169-177.

Chen, P., Wang, C., Lin, D., Li, B., Ye, S., Qu, J., and Wang, W. (2021). Identification of Slc6a19os and SOX11 as Two Novel Essential Genes in Neuropathic Pain Using Integrated Bioinformatic Analysis and Experimental Verification. *Frontiers in Neuroscience* 15.

Dai, D., Wang, J., Jiang, Y., Yuan, L., Lu, Y., Zhang, A., Zou, D., and Chen, X. (2019). Small RNA sequencing reveals microRNAs related to neuropathic pain in rats. *Brazilian Journal of Medical and Biological Research* 52.

Ding, M., Shen, W., and Hu, Y. (2017). The role of miR-539 in the anterior cingulate cortex in chronic neuropathic pain. *Pain Medicine (United States)* 18**,** 2433-2442.

Dong, Y.C., Li, P.F., Ni, Y.H., Zhao, J.J., and Liu, Z.Q. (2014). Decreased MicroRNA-125a-3p Contributes to Upregulation of p38 MAPK in Rat Trigeminal Ganglions with Orofacial Inflammatory Pain. *Plos One* 9.

Genda, Y., Arai, M., Ishikawa, M., Tanaka, S., Okabe, T., and Sakamoto, A. (2013). MicroRNA changes in the dorsal horn of the spinal cord of rats with chronic constriction injury: A TaqMan® Low Density Array study. *International Journal of Molecular Medicine* 31**,** 129-137.

Hori, N., Narita, M., Yamashita, A., Horiuchi, H., Hamada, Y., Kondo, T., Watanabe, M., Igarashi, K., Kawata, M., Shibasaki, M., Yamazaki, M., Kuzumaki, N., Inada, E., Ochiya, T., Iseki, M., Mori, T., and Narita, M. (2016). Changes in the expression of IL-6-Mediated MicroRNAs in the dorsal root ganglion under neuropathic pain in mice. *Synapse* 70**,** 317-324.

Hori, Y., Goto, G., Arai-Iwasaki, M., Ishikawa, M., and Sakamoto, A. (2013). Differential expression of rat hippocampal microRNAs in two rat models of chronic pain. *International Journal of Molecular Medicine* 32**,** 1287-1292.

Li, H.X., Shen, L., Ma, C., and Huang, Y.G. (2013). Differential expression of miRNAs in the nervous system of a rat model of bilateral sciatic nerve chronic constriction injury. *International Journal Of Molecular Medicine* 32**,** 219-226.

Liu, L., Xu, D., Wang, T., Zhang, Y., Yang, X.J., Wang, X.X., and Tang, Y.Y. (2020). Epigenetic reduction of miR-214-3p upregulates astrocytic colony-stimulating factor-1 and contributes to neuropathic pain induced by nerve injury. *Pain* 161**,** 96-108.

Liu, Y., Wang, L., Lao, J., and Zhao, X. (2018). Changes in microRNA expression in the brachial plexus avulsion model of neuropathic pain. *International Journal of Molecular Medicine* 41**,** 1509-1517.

Lu, S., Ma, S.C., Wang, Y.Y., Huang, T., Zhu, Z.H., and Zhao, G.Q. (2017). Mus musculus-microRNA-449a ameliorates neuropathic pain by decreasing the level of KCNMA1 and TRPA1, and increasing the level of TPTE. *Molecular Medicine Reports* 16**,** 353-360.

Rau, C.S., Jeng, J.C., Jeng, S.F., Lu, T.H., Chen, Y.C., Liliang, P.C., Wu, C.J., Lin, C.J., and Hsieh, C.H. (2010). Entrapment neuropathy results in different microRNA expression patterns from denervation injury in rats. *BMC Musculoskeletal Disorders* 11**,** 181-181.

Satyanarayanan, S.K., Shih, Y.H., Wen, Y.R., Palani, M., Lin, Y.W., Su, H.X., Galecki, P., and Su, K.P. (2019). miR-200a-3p modulates gene expression in comorbid pain and depression: Molecular implication for central sensitization. *Brain Behavior And Immunity* 82**,** 230-238.

Von Schack, D., Agostino, M.J., Murray, B.S., Li, Y., Reddy, P.S., Chen, J., Choe, S.E., Strassle, B.W., Li, C., Bates, B., Zhang, L., Hu, H., Kotnis, S., Bingham, B., Liu, W., Whiteside, G.T., Samad, T.A., Kennedy, J.D., and Ajit, S.K. (2011). Dynamic changes in the microRNA expression profile reveal multiple regulatory mechanisms in the spinal nerve ligation model of neuropathic pain. *PLoS ONE* 6.

Wilkerson, J.L., Jiang, J., Felix, J.S., Bray, J.K., Da Silva, L., Gharaibeh, R.Z., Mcmahon, L.R., and Schmittgen, T.D. (2020). Alterations in mouse spinal cord and sciatic nerve microRNAs after the chronic constriction injury (CCI) model of neuropathic pain. *Neuroscience Letters* 731.

Zhou, J., Xiong, Q.M., Chen, H.T., Yang, C.X., and Fan, Y.L. (2017). Identification of the Spinal Expression Profile of Non-coding RNAs Involved in Neuropathic Pain Following Spared Nerve Injury by Sequence Analysis. *Frontiers In Molecular Neuroscience* 10.

Table S3. Expression profiles of miRNA in disease-induced NP models

| **Article, Year** | **Country** | **Expression** | **miRNAs** | **Experimental models** | **Method** |
| --- | --- | --- | --- | --- | --- |
| Bali et al., 2013 ([Bali et al., 2013](#_ENREF_1)) | Germany | up | mmu-miR-544-3p, mmu-miR-133a-5p, mmu-miR-154-3p, mmu-miR-377-3p, mmu-miR-141-3p, solexa-2012-235, mmu-miR-142-5p, solexa-4179-110, mmu-miR-380-3p, mmu-miR-130b-3p, mmu-miR-16-1-3p, mmu-miR-15b-3p, mmu-miR-376c-5p, solexa-5306-86, mmu-miR-1224-5p, mmu-miR-1188-5p, mmu-miR-291b-5p, solexa-1278-371, mmu-miR-702-3p, mmu-miR-370-3p, mmu-miR-669c-5p, mmu-miR-877-5p, mmu-miR-874-3p, mmu-miR-298-5p, mmu-miR-296-5p, mmu-miR-1198-5p, mmu-miR-31-5p | L3-L4 DRGs from bone metastatic pain mice | Illumina Mouse Sentrix-6 beadchip arrays |
|  |  | down | mmu-miR-200a-3p, mmu-miR-34b-5p, solexa-5067-90, mmu-miR-376c-3p, mmu-miR-669j, mmu-miR-380-5p, mmu-miR-34c-5p, mmu-miR-181a-1-3p, mmu-miR-582-3p, mmu-miR-369-3p, mmu-miR-1a-3p, mmu-miR-496a-3p, mmu-miR-499-5p, mmu-miR-483-3p, mmu-miR-423-5p, mmu-miR-323-5p, mmu-miR-760-3p, mmu-miR-466i-3p, mmu-miR-681, solexa-403-1161, mmu-miR-466f-3p, mmu-miR-877-3p, mmu-miR-466g, mmu-miR-673-5p, mmu-miR-466d-3p, mmu-miR-714, mmu-miR-218-1-3p, mmu-miR-483-5p, mmu-miR-467a-3p |  |  |
| Gong et al., 2014 ([Gong et al., 2014](#_ENREF_2)) | China | up | mmu-miR-3965, mmu-miR-3063-5p, mmu-miR-466n-5p, mmu-miR-505-5p, mmu-miR-196a-2-3p, mmu-miR-5710, mmu-miR-466a-5p, mmu-miR-466b-5p, mmu-miR-3473a, mmu-miR-3060-5p, mmu-miR-122-5p, mmu-miR-466p-5p, mmu-miR-187-3p, mmu-miR-128-1-5p, mmu-miR-3074-2-3p, mmu-miR-210-3p, mmu-miR-3475, mmu-miR-194-1-3p, mmu-miR-27a-5p, mmu-miR-667-3p, mmu-miR-98-5p | SDH from STZ-induced DNP mice | Microarray |
|  |  | down | mmu-miR-190a-5p, mmu-miR-590-3p, mmu-miR-5124a, mmu-miR-302b-3p, mmu-miR-467e-3p, mmu-miR-467g, mmu-miR-669a-3p, mmu-miR-467d-3p, mmu-miR-669a-3-3p, mmu-miR-451a, mmu-miR-669f-3p, mmu-miR-669e-3p, mmu-miR-466i-3p, mmu-miR-568, mmu-miR-669p-3p, mmu-miR-184-5p, mmu-miR-466g, mmu-miR-466q, mmu-miR-669b-3p, mmu-miR-467b-3p, mmu-miR-6360 |  |  |

Abbreviations: DNP: diabetic neuropathic pain; DRG: dorsal root ganglion; NP: neuropathic pain; STZ: streptozotocin.

References

Bali, K.K., Selvaraj, D., Satagopam, V.P., Lu, J., Schneider, R., and Kuner, R. (2013). Genome-wide identification and functional analyses of microRNA signatures associated with cancer pain. *Embo Molecular Medicine* 5**,** 1740-1758.

Gong, Q., Lu, Z., Huang, Q., Ruan, L., Chen, J., Liang, Y., Wang, H., Yue, Y., and Feng, S. (2014). Altered microRNAs expression profiling in mice with diabetic neuropathic pain. *Biochemical and Biophysical Research Communications*.

Table S4. Experimentally verified miRNAs in NP surgical models

| **Article, Year** | **Country** | **Models** | **Animals** | **Region** | **miRNAs** | **Expression change** | **Target gene(s)** | **Functions** |
| --- | --- | --- | --- | --- | --- | --- | --- | --- |
| Aldrich et al., 2009 ([Aldrich et al., 2009](#_ENREF_1)) | USA | SNL | rats | DRG | miR-96, miR-183, miR-182 | down | NR | NR |
| Favereaux et al., 2011 ([Favereaux et al., 2011](#_ENREF_14)) | France | SNL | rats | dorsal spinal cord | miR-103 | down | Ca_V_1.2 | Neuronal excitability |
| Imai et al., 2011 ([Imai et al., 2011](#_ENREF_17)) | Japan | partial sciatic nerve ligation | mice | N.Acc. Neurons | miR-200b, miR-429 | down | DNMT3a | Neuronal adaptivity |
| Sakai et al., 2013 ([Sakai et al., 2013](#_ENREF_46)) | Japan | SNL, CCI | rats | L5 DRG | miR-7a | down | Na_V_b2 | Neuronal excitability |
| Sakai et al., 2013 ([Sakai and Suzuki, 2013](#_ENREF_47)) | Japan | SNL, CCI | rats | L5 DRG | miR-21 | up | NR | Neuroinflammation |
| Shi et al., 2013 ([Shi et al., 2013](#_ENREF_50)) | China | SNL | rats | L5 SDH, microglia | miR-195 | up | ATG14 | Neuroinflammation |
| Chen et al., 2014 ([Chen et al., 2014](#_ENREF_8)) | China | CCI | rats | L4-L6 DRG | miR-96 | down | Na_V_1.3 | Neuronal excitability |
| Dong et al., 2014 ([Dong et al., 2014](#_ENREF_11)) | China | CFA-induced prosopalgia | rats | TGs | miR-125a-3p | down | p38 MAPK | Neuroinflammation |
| Lin et al., 2014 ([Lin et al., 2014](#_ENREF_30)) | China (Taiwan) | SNL | rats | L5 DRG | miR-183 | down | Na_V_1.3, BDNF | Neuronal excitability |
| Li et al., 2015 ([Li et al., 2015](#_ENREF_24)) | China | bCCI | rats | L4-L6 SDH, PC12 cells | miR-203 | down | Rap1A | Neuronal plasticity |
| Liu et al., 2015 ([Liu et al., 2015](#_ENREF_33)) | China | bCCI | rats | microglia | miR-155 | up | SGK3 | Neuroinflammation |
| Lu et al., 2015 ([Lu et al., 2015](#_ENREF_36)) | China | SNL | mice | spinal cord, astrocytes | miR-146a-5p | up | TRAF6 | Neuroinflammation |
| Neumann et al., 2015 ([Neumann et al., 2015](#_ENREF_40)) | Germany | CCI | rats | sciatic nerve | miR-1 | down | BDNF, Cx43 | Neuroinflammation |
| Tan et al., 2015 ([Tan et al., 2015](#_ENREF_57)) | China | CCI | rats | spinal cord, microglia | miR-155 | up | SOCS1 | Neuroinflammation |
| Wang et al., 2015 ([Wang et al., 2015](#_ENREF_62)) | China | bCCI | rats | spinal cord | miR-19a | up | SOCS1 | Neuroinflammation |
| Zhang et al., 2015 ([Zhang et al., 2015](#_ENREF_81)) | China | CCI | rats | DRG, DRG neurons | miR‑141 | down | HMGB1 | Neuroinflammation |
| Chen et al., 2016 ([Chen et al., 2016](#_ENREF_7)) | China | bCCI | rats | L4-L6 dorsal spinal cord | miR-30a | down | SOCS1 | Neuroinflammation |
| Jiang et al., 2016 ([Jiang et al., 2016](#_ENREF_20)) | China | SNL | mice | L5 spinal cord, SDH neurons, astrocytes | miR-186-5p | down | CXCL13, CXCR5 | Neuroinflammation |
| Leinders et al., 2016 ([Leinders et al., 2016](#_ENREF_23)) | USA | SNI | rats | L4-L5 DRG, dorsal spinal cord , microglia | miR-132-3p | up | GluA1, GluA2 | Neuronal plasticity |
| Li et al., 2016 ([Li and Zhao, 2016](#_ENREF_26)) | China | CCI | rats | spinal cord, microglia | miR-218 | up | SOCS3 | Neuroinflammation |
| Manners et al., 2016 ([Manners et al., 2016](#_ENREF_37)) | USA | SNI | mice | L4-L6 DRG, Neuro 2a cells | miR-126 | down | Dnmt1, Vegfa | DNA methylation |
| Pang et al., 2016 ([Pang et al., 2016](#_ENREF_42)) | China | CCI | rats | spinal cord | miR-145 | down | RREB1, p-AKT | Neuroinflammation |
| Shao et al., 2016 ([Shao et al., 2016](#_ENREF_48)) | China | SNI | rats | DRG, PC12 cells | miR-30b | down | Na_V_1.7 | Neuronal excitability |
| Xia et al., 2016 ([Xia et al., 2016](#_ENREF_67)) | China | CCI | rats | spinal cord, microglia | miR-221 | up | SOCS1 | Neuroinflammation |
| Yang et al., 2016 ([Yang et al., 2016](#_ENREF_76)) | China | SNL, CCI | rats | DRG, PC12 cells | miR-206 | down | RASA1 | Neuronal plasticity |
| Karl et al., 2017 ([Karl et al., 2017](#_ENREF_22)) | Germany | SNI | mice | sural nerve | miR-21 | up | NR | Neuroinflammation |
| Lu et al., 2017 ([Lu et al., 2017](#_ENREF_35)) | China | SNI | mice | DRG, DRG neurons | mmu-miR-449a | down | TRPA1, KCNMA1, TPTE | Neuronal excitability |
| Peng et al., 2017 ([Peng et al., 2017](#_ENREF_43)) | Sweden | SNI | mice | L4-L6 DRG | miR-183, miR-96, miR-182 | down | Cacna2d1, Cacna2d2 | Neuronal excitability |
| Sakai et al., 2017 ([Sakai et al., 2017](#_ENREF_45)) | Japan | SNL | rats | L5 DRG | miR-17, miR-18a, miR-19a, miR-19b, miR-20a, miR-92a | up | K_V_1.1, K_V_3.4, K_V_4.3 | Neuronal excitability |
| Simwoli et al., 2017 ([Simeoli et al., 2017](#_ENREF_52)) | UK | SNI | mice | L5 DRG | miR-21-5p | up | NR | Neuroimmune |
| Su et al., 2017 ([Su et al., 2017](#_ENREF_53)) | China | SNL | rats | spinal cord, DRG neurons | miR-30b | down | Na_V_1.3 | Neuronal excitability |
| Sun et al., 2017 ([Sun et al., 2017](#_ENREF_55)) | China | CCI | rats | DRG, PC12 cells | miR-206 | down | BDNF | Neuroinflammation |
| Xie et al., 2017 ([Xie et al., 2017](#_ENREF_69)) | China | CCI | rats | L4-L6 SDH, PC12 cells | miR-183 | down | mTOR, VEGF | Neuroinflammation |
| Xu et al., 2017 ([Xu et al., 2017](#_ENREF_70)) | USA | SNL | rats | L5 DRG, DRG neurons | miR-143 | down | Dnmt3a | DNA methylation |
| Yan et al., 2017 ([Yan et al., 2017](#_ENREF_73)) | China | bCCI | rats | L4-L6 dorsal spinal cords, microglia | miR-93 | down | STAT3 | Neuroinflammation |
| Zhou et al., 2017 ([Zhou et al., 2017](#_ENREF_94)) | China | CCI | mice | spinal cord | miR-182-5p | down | ephb1 | Neuronal excitability |
| Bao et al., 2018 ([Bao et al., 2018](#_ENREF_2)) | China | bCCI | rats | L4-L6 dorsal spinal cords, microglia, HEK‐293T cells | miR-28-5p | down | Zeb1 | Neuroinflammation |
| Cai et al., 2018 ([Cai et al., 2018](#_ENREF_6)) | China | SNI | rats | L4-L5 DRG, DRG neurons | miR-182 | down | Na_V_1.7 | Neuronal excitability |
| Chen et al., 2018 ([Chen et al., 2018](#_ENREF_9)) | China | CFA-induced prosopalgia | mice | TGs | miR-186 | down | NLRP3 | Neuroinflammation |
| Ji et al., 2018 ([Ji et al., 2018](#_ENREF_18)) | China | bCCI | rats | L4-L6 dorsal spinal cords, microglia | miR-150 | down | TLR5 | Neuroinflammation |
| Li et al., 2018 ([Li et al., 2018](#_ENREF_28)) | China | sciatic nerve transection | rats | sciatic nerves | miR-146b | down | KLF7 | Neuronal plasticity |
| Liu et al., 2018 ([Liu et al., 2018](#_ENREF_34)) | China | sciatic nerve transection | rats | sciatic nerves, RSC96 cells | miR-1b | down | NDRG3 | Neuronal plasticity |
| Pan et al., 2018 ([Pan et al., 2018](#_ENREF_41)) | China | partial sciatic nerve ligation | mice | L3–L5 SDH | miR-23a | down | CXCR4 | Neuroinflammation |
| Shi D. et al., 2018 ([Shi et al., 2018a](#_ENREF_49)) | China | CCI | rats | L4-L5 DRG | miR-183-5p | down | TREK-1 | Neuronal excitability |
| Shi J. et al., 2018 ([Shi et al., 2018b](#_ENREF_51)) | China | CCI | rats | L5 DRG, PC12 cells | miR-145 | down | Akt3, mTOR | Neuroinflammation |
| Wang et al., 2018 ([Wang et al., 2018](#_ENREF_65)) | China | CCI | rats | L4-L6 DRG, SDH | miR-146a-5p | up | IRAK1, TRAF6 | Neuroinflammation |
| Tramullas et al., 2018 ([Tramullas et al., 2018](#_ENREF_60)) | Spain | SNI | mice | spinal cord, DRG | miR-30c-5p | up | TGF-β | Neural plasticity |
| Yan et al., 2018 ([Yan et al., 2018a](#_ENREF_72)) | China | SNL | rats | SDH, microglia | miR-32-5p | up | Dusp5 | Neuroinflammation |
| Yan X. et al., 2018 ([Yan et al., 2018b](#_ENREF_74)) | China | CCI | rats | spinal cord, microglia, HEK-293T cells | miR-200b, miR-429 | down | ZEB1 | Neuroinflammation |
| Zhan et al., 2018 ([Zhan et al., 2018](#_ENREF_79)) | China | CCI | rats | spinal cord, microglia, HEK-294T cells | miR-381 | down | HMGB1, CXCR4 | Neuroinflammation |
| Zhang et al., 2018 ([Zhang et al., 2018a](#_ENREF_87)) | China | SNL | mice | DRG, DRG neurons | miR‑142‑3p | down | HMGB1 | Neuroinflammation |
| Zhang Y. et al. 2018 ([Zhang et al., 2018b](#_ENREF_89)) | China | CCI | rats | L4-L6 SDH, microglia, HEK‐293T cells | miR-26a-5p | down | MAPK6 | Neuroinflammation |
| Zhang Z et al., 2018 ([Zhang et al., 2018c](#_ENREF_90)) | China | SNL | mice, mice | DRG, DRG neurons, HEK293 cells | miR-21 | up | TLR8 | Neuronal excitability |
| Zhu et al., 2018 ([Zhu et al., 2018](#_ENREF_96)) | China | sciatic nerve transection | rats | L4-L6 DRGs, DRG neuron, Schwann cells | miR-129 | down | IGF-1 | Neuronal plasticity |
| Brandenburger et al., 2019 ([Brandenburger et al., 2019](#_ENREF_3)) | Germany | SNL | rats | L4-L6 DRGs | miR-34a | down | VAMP-2 | Neuroinflammation |
| Cai et al., 2019 ([Cai et al., 2019](#_ENREF_5)) | China | bCCI | rats | L4-L6 dorsal spinal cords | miR-150 | down | AKT3 | Neuroinflammation |
| Chu et al., 2019 ([Chu et al., 2019](#_ENREF_10)) | China | bCCI | rats | L4-L6 dorsal spinal cords, microglia | miR-448 | up | SIRT1 | Neuroinflammation |
| Fang et al., 2019 ([Fang et al., 2019a](#_ENREF_12)) | China | bCCI | rats | L4-L6 SDH, PC12 cells | miR-202 | down | Rap1A | Neuronal plasticity |
| Fang X. et al., 2019 ([Fang et al., 2019b](#_ENREF_13)) | China | CCI | rats | spinal cord | miR-1906 | down | TLR4, mTOR, Akt | Neuroinflammation |
| Gao et al., 2019 ([Gao et al., 2019](#_ENREF_15)) | China | CCI | rats | L4-L5 spinal cord, microglia | miR-340-5p | down | Rap1A | Neuronal plasticity |
| Ji et al., 2019 ([Ji et al., 2019](#_ENREF_19)) | China | bCCI | rats | SDH, microglia | miR-134-5p | down | Twist1 | Neuroinflammation |
| Jiang et al., 2019 ([Jiang et al., 2019](#_ENREF_21)) | China | bCCI | rats | SDH, PC12 cells | miR-217 | up | TLR5 | Neuroinflammation |
| Li et al., 2019 ([Li et al., 2019](#_ENREF_27)) | China | CCI | rats | spinal cord | miR-15a, miR-16 | up | GRK2 | Neuroinflammation |
| Tozaki-Saitoh et al., 2019 ([Tozaki-Saitoh et al., 2019](#_ENREF_59)) | Japan | SNL | mice | L4 spinal cords, microglia | mir-152 | down | MafB | Neuronal excitability |
| Wang et al., 2019 ([Wang et al., 2019](#_ENREF_64)) | China | CCI-IoN | rats | caudal medulla | miR-195 | up | Patched1 | Neuronal proliferation |
| Wen et al., 2019 ([Wen et al., 2019](#_ENREF_66)) | China | CCI | rats | L4-L5 DRG | miR-206-3p | down | HDAC4 | Neuron regeneration |
| Xu et al., 2019 ([Xu et al., 2019](#_ENREF_71)) | China | CCI | mice | L4-L6 dorsal spinal cords | miR-34c | down | NLRP3 | Neuroinflammation |
| Yang et al., 2019 ([Yang et al., 2019](#_ENREF_75)) | China | SNL | rats | L4-L5 DRG | miR-7a | down | NEFL, STAT3 | Neuroinflammation |
| You et al., 2019 ([You et al., 2019](#_ENREF_78)) | China | CCI | rats | L4-L6 dorsal spinal cords, microglia, PC12 cell | miR-20b-5p | down | Akt3 | Neuroinflammation |
| Zhang et al., 2019 ([Zhang et al., 2019a](#_ENREF_85)) | China | bCCI | rats | L4-L6 dorsal spinal cords, microglia, HEK‐293T | miR-124-3p | down | EZH2 | Neuroinflammation |
| Zhang Y. et al., 2019 ([Zhang et al., 2019b](#_ENREF_88)) | China | bCCI | rats | L5-L6 dorsal spinal cords, HEK‐293T cells | miR-98 | down | HMGA2 | Neuroinflammation |
| Zhong et al., 2019 ([Zhong et al., 2019a](#_ENREF_91)) | China | bCCI | rats | L4-L6 dorsal spinal cords, microglia, HEK‐293T | miR-98 | down | STAT3 | Neuroinflammation |
| Zhong L. et al., 2019 ([Zhong et al., 2019b](#_ENREF_92)) | China | CCI | rats | L4-L6 dorsal spinal cords | miR-21-5p | down | TIMP3, CCL1 | Neuroinflammation |
| Zhu et al., 2019 ([Zhu et al., 2019](#_ENREF_95)) | China | SNI | mice | L4-L6 SDH | miR138 | down | NF-Κв | Neuroinflammation |
| Cai et al.,2020 ([Cai et al., 2020](#_ENREF_4)) | China | CCI | rats | spinal cords, microglia | miR-15a | down | AKT3 | Neuroinflammation |
| Huang et al., 2020 ([Huang and Wang, 2020](#_ENREF_16)) | China | bCCI | rats | L4-L6 dorsal spinal cords, microglia | miR-183 | down | MAP3K4 | Neuroinflammation |
| Li et al., 2020 ([Li et al., 2020](#_ENREF_25)) | China | CCI | rats | L5 DRG | miR-140 | down | S1PR1 | Neuroinflammation |
| Liu et al., 2020 ([Liu et al., 2020](#_ENREF_32)) | China | SNL | rats | L4-L6 SDH, microglia, HEK‐293T | miR-214-3p | down | CSF1 | DNA methylation |
| Miao et al., 2020 ([Miao et al., 2020](#_ENREF_38)) | China | CCI | rats | SDH, microglia | miR-183 | down | TXNIP | Neuroinflammation |
| Tan et al., 2020 ([Tan et al., 2020](#_ENREF_56)) | China | CCI | rats | L4-L7 SDH, microglia | miR-30a-3p | down | EP300 | Neuroinflammation |
| Tian et al., 2020 ([Tian et al., 2020](#_ENREF_58)) | China | bCCI | rats | L4-L6 SDH, microglia | miR-129-5p | down | HMGB1 | Neuroinflammation |
| Wang et al., 2020 ([Wang and Li, 2020](#_ENREF_63)) | China | CCI | rats | DRG, microglia | miR-216a-5p | down | KDM3A | Neuroinflammation |
| Xie et al., 2020 ([Xie et al., 2020](#_ENREF_68)) | China | CCI | rats | L4-L6 SDH, microglia | miR-101 | down | mTOR | Neuroinflammation |
| Ye et al., 2020 ([Ye et al., 2020](#_ENREF_77)) | China | CCI | rats | DRG neurons | miR-384-5p | down | Nav1.3 | Neuronal excitability |
| Zhang et al., 2020 ([Zhang et al., 2020a](#_ENREF_82)) | China | bCCI | rats | L4-L6 dorsal spinal cord, HEK‐293T cells | miR-194 | down | FOXA1 | Neuroinflammation |
| Zhang X. et al., 2021 ([Zhang et al., 2020c](#_ENREF_84)) | China | CCI | rats | L4-L6 SDH, microglia | miR-128-3p | down | ZEB1 | Neuroinflammation |
| Zhang X. et al., 2020 ([Zhang et al., 2020b](#_ENREF_83)) | China | CCI | mice | L4-L5 spinal cord, DRG | miR-144 | down | RASA1 | Neuroinflammation |
| Zhou et al., 2020 ([Zhou et al., 2020](#_ENREF_93)) | China | CCI | rats | L4-L6 SDH, DRG | miR-547 | down | IL-33, ST2 | Neuroinflammation |
| Li et al., 2021 ([Li et al., 2021](#_ENREF_29)) | China | CCI | rats | L4-L6 sciatic nerve, 293T cells | miR‑142‑3p | up | AC9 | Neuroinflammation |
| Lin et al., 2020 ([Lin et al., 2020](#_ENREF_31)) | China | CCI | rats | L4-L6 spinal cord, microglia | miR-665 | up | SOCS1 | Neuroinflammation |
| Mo et al., 2020 ([Mo et al., 2020](#_ENREF_39)) | China | bCCI | rats | L4-L6 dorsal spinal cords, DRG neurons | miR-34c-5p | up | SIRT1 | Neuroinflammation |
| Qiu et al., 2020 ([Qiu et al., 2020](#_ENREF_44)) | China | CCI | rats | L4-L6 SDH, microglia | miR-101 | up | MKP-1 | Neuroinflammation |
| Sun et al., 2021 ([Sun et al., 2021](#_ENREF_54)) | China | SNI | mice | L4-L6 SDH, DRG | miR-96 | down | Na_V_1.7, Na_V_1.8 | Neuronal excitability |
| Wan et al., 2021 ([Wan et al., 2021](#_ENREF_61)) | China | CCI | mice | spinal cord, HEK-293T cell | miR-122-5p | down | PDK4 | Neuroinflammation |
| Zhang et al., 2021 ([Zhang et al., 2021a](#_ENREF_80)) | China | CCI | rats | L4-L6 SDH, DRG | miR-137 | up | Kv1.2 | Neuronal excitability |
| Zhang Y. et al., 2021 ([Zhang et al., 2021b](#_ENREF_86)) | China | SNI | mice | L4-L5 DRGs, DRG neurons | miR-23a | up | A20 | Inflammatory macrophage polarization |

Abbreviations: AC9: adenylate cyclase 9; bCCI: bilateral chronic constriction sciatic nerve injury; BDNF: brain-derived neurotrophic factor; Cacna2d1, Cacna2d2: voltage-gated calcium channel subunits α2δ-1 and α2δ-2; CCI: chronic constriction sciatic nerve injury; CCI-IoN: infraorbital nerve chronic constriction injury; CCL1: chemokines C-C motif ligand 1; CFA: Freund’s adjuvant; CSF1: colony-stimulating factor-1; CXCL13: C-X-C motif chemokine 13; CXCR4: Chemokine CXC receptor 4; Dnmt1: DNA methyltransferase 1; DNMT3a: DNA methyltransferase 3a; DRG: dorsal root ganglion; Dusp5: Dual-specificity phosphatase 5; EP300: E-cadherin transcriptional activator; ephb1: ephrin type-b receptor 1; FOXA1: Forkhead box protein A1; GRK2: G protein-coupled receptor kinase 2; HDAC4: histone deacetylase 4; HMGA2: high mobility group A2; HMGB1, high-mobility group box 1; IGF-1: insulin-like growth factor-1; IL-33: Interleukin-33; IRAK1: interleukin-1 receptor-associated kinase 1; KCNMA1: calcium‑activated potassium channel subunit α‑1; KLF7: Kruppel like factor 7; MKP-1: mitogen-activated protein kinase phosphatase 1; mTOR: mammalian target of rapamycin; N.Acc: nucleus accumbens; Na_V_b2: voltage-gated sodium channel b2 subunit; NDRG3: N-myc downstream-regulated gene 3; NEFL: neurofilament light polypeptide; NF-κВ: nuclear factor-κВ; NLRP3, nucleotide binding domain-like receptor protein 3; NR: not reported; p-AKT: phosphorylated-protein kinase B; Rap1A: Ras-related protein 1A; RASA1: RAS p21 protein activator 1; RREB1: ras responsive element binding protein 1; S1PR1: Sphingosine-1-phosphate receptor 1; SDH: spinal dorsal horn; SGK3: serum and glucocorticoid regulated protein kinase 3; SIRT1: sirtuin 1; SNI: spared nerve injury; SNL: spinal nerve ligation; SOCS1: suppressor of cytokine signaling 1; ST2: suppressor of tumorigenicity 2; STAT3: signal transducer and activator of transcription 3; TGF-β: a transforming growth factor-β; TGs: trigeminal ganglions; TIMP3: tissue inhibitor of metalloproteinase-3; TLR5: Toll-like receptor 5; TPTE: transmembrane phosphatase with tension homology; TRAF6: TNF receptor associated factor-6; TRPA1: transient receptor potential cation channel subfamily A member 1; TXNIP: thioredoxin interacting protein; VAMP-2: vesicle-associated membrane protein 2; VEGF: vascular endothelial growth factor; Vegfa: vascular endothelial growth factor A; Zeb1: zinc finger E-box-binding homeobox 1.

References

Aldrich, B.T., Frakes, E.P., Kasuya, J., Hammond, D.L., and Kitamoto, T. (2009). Changes in expression of sensory organ-specific microRNAs in rat dorsal root ganglia in association with mechanical hypersensitivity induced by spinal nerve ligation. *Neuroscience* 164**,** 711-723.

Bao, Y.F., Wang, S.H., Xie, Y.S., Jin, K.H., Bai, Y.T., and Shan, S.G. (2018). MiR-28-5p relieves neuropathic pain by targeting Zeb1 in CCI rat models. *Journal Of Cellular Biochemistry* 119**,** 8555-8563.

Brandenburger, T., Johannsen, L., Prassek, V., Kuebart, A., Raile, J., Wohlfromm, S., Kohrer, K., Huhn, R., Hollmann, M.W., and Hermanns, H. (2019). MiR-34a is differentially expressed in dorsal root ganglia in a rat model of chronic neuropathic pain. *Neuroscience Letters* 708.

Cai, L.X., Liu, X.F., Guo, Q.C., Huang, Q., Zhang, Q., and Cao, Z.H. (2020). MiR-15a attenuates peripheral nerve injury-induced neuropathic pain by targeting AKT3 to regulate autophagy. *Genes & Genomics* 42**,** 77-85.

Cai, W., Zhang, Y., Liu, Y., Liu, H., Zhang, Z., and Su, Z. (2019). Effects of miR-150 on neuropathic pain process via targeting AKT3. *Biochemical and Biophysical Research Communications* 517**,** 532-537.

Cai, W., Zhao, Q., Shao, J., Zhang, J., Li, L., Ren, X., Su, S., Bai, Q., Li, M., Chen, X., Wang, J., Cao, J., and Zang, W. (2018). MicroRNA-182 Alleviates Neuropathic Pain by Regulating Nav1.7 Following Spared Nerve Injury in Rats. *Scientific reports* 8**,** 16750.

Chen, H., Wang, Y., Xu, Y., and Wang, G.N. (2016). Overexpression of miR-30a attenuates neuropathic pain by targeting SOCS1 in rats with chronic constriction injury. *International Journal Of Clinical And Experimental Pathology* 9**,** 1258-1266.

Chen, H.P., Zhou, W., Kang, L.M., Yan, H., Zhang, L., Xu, B.H., and Cai, W.H. (2014). Intrathecal miR-96 Inhibits Nav1.3 Expression and Alleviates Neuropathic Pain in Rat Following Chronic Construction Injury. *Neurochemical Research* 39**,** 76-83.

Chen, M.L., Lin, K., and Lin, S.K. (2018). NLRP3 inflammasome signaling as an early molecular response is negatively controlled by miR-186 in CFA-induced prosopalgia mice. *Brazilian Journal of Medical and Biological Research* 51.

Chu, Y.C., Ge, W.P., and Wang, X. (2019). MicroRNA-448 modulates the progression of neuropathic pain by targeting sirtuin 1. *Experimental And Therapeutic Medicine* 18**,** 4665-4672.

Dong, Y.C., Li, P.F., Ni, Y.H., Zhao, J.J., and Liu, Z.Q. (2014). Decreased MicroRNA-125a-3p Contributes to Upregulation of p38 MAPK in Rat Trigeminal Ganglions with Orofacial Inflammatory Pain. *Plos One* 9.

Fang, B., Wei, L., Dong, K., Niu, X., Sui, X., and Zhang, H. (2019a). miR-202 modulates the progression of neuropathic pain through targeting RAP1A. *Journal of Cellular Biochemistry* 120**,** 2973-2982.

Fang, X., Zhou, H., Huang, S., and Liu, J. (2019b). MiR-1906 attenuates neuropathic pain in rats by regulating the TLR4/mTOR/ Akt signaling pathway. *Translational Neuroscience* 10**,** 175-179.

Favereaux, A., Thoumine, O., Bouali-Benazzouz, R., Roques, V., Papon, M.A., Salam, S.A., Drutel, G., Léger, C., Calas, A., Nagy, F., and Landry, M. (2011). Bidirectional integrative regulation of Cav1.2 calcium channel by microRNA miR-103: Role in pain. *EMBO Journal* 30**,** 3830-3841.

Gao, L., Pu, X.H., Huang, Y.J., and Huang, J. (2019). MicroRNA-340-5p relieved chronic constriction injury-induced neuropathic pain by targeting Rap1A in rat model. *Genes & Genomics* 41**,** 713-721.

Huang, L.L., and Wang, L. (2020). Upregulation of miR-183 represses neuropathic pain through inhibiton of MAP3K4 in CCI rat models. *Journal Of Cellular Physiology* 235**,** 3815-3822.

Imai, S., Saeki, M., Yanase, M., Horiuchi, H., Abe, M., Narita, M., Kuzumaki, N., Suzuki, T., and Narita, M. (2011). Change in microRNAS associated with neuronal adaptive responses in the nucleus accumbens under neuropathic pain. *Journal of Neuroscience* 31**,** 15294-15299.

Ji, L.J., Shi, J., Lu, J.M., and Huang, Q.M. (2018). MiR-150 alleviates neuropathic pain via inhibiting toll-like receptor 5. *Journal of Cellular Biochemistry* 119**,** 1017-1026.

Ji, L.J., Su, J., Xu, A.L., Pang, B., and Huang, Q.M. (2019). MiR-134-5p attenuates neuropathic pain progression through targeting Twist1. *Journal of Cellular Biochemistry* 120**,** 1694-1701.

Jiang, B.C., Cao, D.L., Zhang, X., Zhang, Z.J., He, L.N., Li, C.H., Zhang, W.W., Wu, X.B., Berta, T., Ji, R.R., and Gao, Y.J. (2016). CXCL13 drives spinal astrocyte activation and neuropathic pain via CXCR5. *Journal of Clinical Investigation* 126**,** 745-761.

Jiang, W., Wang, Q., Yu, X., Lu, T., and Zhang, P. (2019). MicroRNA-217 relieved neuropathic pain through targeting toll-like receptor 5 expression. *Journal of Cellular Biochemistry* 120**,** 3009-3017.

Karl, F., Griesshammer, A., Uceyier, N., and Sommer, C. (2017). Differential Impact of miR-21 on Pain and Associated Affective and Cognitive Behavior after Spared Nerve Injury in B7-H1 ko Mouse. *Frontiers In Molecular Neuroscience* 10.

Leinders, M., çeyler, N., Pritchard, R.A., Sommer, C., and Sorkin, L.S. (2016). Increased miR-132-3p expression is associated with chronic neuropathic pain. *Experimental Neurology* 283**,** 276-286.

Li, H., Huang, Y., Ma, C., Yu, X., Zhang, Z., and Shen, L. (2015). MiR-203 involves in neuropathic pain development and represses Rap1a expression in nerve growth factor differentiated neuronal PC12 cells. *Clin J Pain* 31**,** 36-43.

Li, J., Zhu, Y., Ma, Z., Liu, Y., Sun, Z., and Wu, Y. (2020). miR-140 ameliorates neuropathic pain in CCI rats by targeting S1PR1. *Journal of Receptors and Signal Transduction*.

Li, L.Y., and Zhao, G.Q. (2016). Downregulation of microRNA-218 relieves neuropathic pain by regulating suppressor of cytokine signaling 3. *International Journal Of Molecular Medicine* 37**,** 851-858.

Li, T., Wan, Y., Sun, L., Tao, S., Chen, P., Liu, C., Wang, K., Zhou, C., and Zhao, G. (2019). Inhibition of microRNA-15a/16 expression alleviates neuropathic pain development through upregulation of G protein-coupled receptor kinase 2. *Biomolecules and Therapeutics* 27**,** 414-422.

Li, W.Y., Zhang, W.T., Cheng, Y.X., Liu, Y.C., Zhai, F.G., Sun, P., Li, H.T., Deng, L.X., Zhu, X.F., and Wang, Y. (2018). Inhibition of KLF7-Targeting MicroRNA 146b Promotes Sciatic Nerve Regeneration. *Neurosci Bull* 34**,** 419-437.

Li, X., Wang, S., Yang, X., and Chu, H. (2021). miR-142-3p targets AC9 to regulate sciatic nerve injury-induced neuropathic pain by regulating the cAMP/AMPK signalling pathway. *International Journal of Molecular Medicine* 47**,** 561-572.

Lin, C.R., Chen, K.H., Yang, C.H., Huang, H.W., and Sheen-Chen, S.M. (2014). Intrathecal miR-183 delivery suppresses mechanical allodynia in mononeuropathic rats. *European Journal of Neuroscience* 39**,** 1682-1689.

Lin, Y., Li, M., Rao, G., Zhang, W., and Chen, X. (2020). Inhibition of miR-665 alleviates neuropathic pain by targeting SOCS1. *Tropical Journal of Pharmaceutical Research* 19**,** 1591-1597.

Liu, L., Xu, D., Wang, T., Zhang, Y., Yang, X.J., Wang, X.X., and Tang, Y.Y. (2020). Epigenetic reduction of miR-214-3p upregulates astrocytic colony-stimulating factor-1 and contributes to neuropathic pain induced by nerve injury. *Pain* 161**,** 96-108.

Liu, S.X., Zhu, B., Sun, Y., and Xie, X.F. (2015). miR-155 modulates the progression of neuropathic pain through targeting SGK3. *International Journal Of Clinical And Experimental Pathology* 8**,** 14374-14382.

Liu, Y.P., Xu, P., Guo, C.X., Luo, Z.R., Zhu, J., Mou, F.F., Cai, H., Wang, C., Ye, X.C., Shao, S.J., and Guo, H.D. (2018). miR-1b overexpression suppressed proliferation and migration of RSC96 and increased cell apoptosis. *Neuroscience Letters* 687**,** 137-145.

Lu, S., Ma, S.C., Wang, Y.Y., Huang, T., Zhu, Z.H., and Zhao, G.Q. (2017). Mus musculus-microRNA-449a ameliorates neuropathic pain by decreasing the level of KCNMA1 and TRPA1, and increasing the level of TPTE. *Molecular Medicine Reports* 16**,** 353-360.

Lu, Y., Cao, D.L., Jiang, B.C., Yang, T., and Gao, Y.J. (2015). MicroRNA-146a-5p attenuates neuropathic pain via suppressing TRAF6 signaling in the spinal cord. *Brain, Behavior, and Immunity* 49**,** 119-129.

Manners, M.T., Ertel, A., Tian, Y., and Ajit, S.K. (2016). Genome-wide redistribution of MeCP2 in dorsal root ganglia after peripheral nerve injury. *Epigenetics Chromatin* 9**,** 23.

Miao, J., Zhou, X., Ji, T., and Chen, G. (2020). NF-κB p65-dependent transcriptional regulation of histone deacetylase 2 contributes to the chronic constriction injury-induced neuropathic pain via the microRNA-183/TXNIP/NLRP3 axis. *Journal of Neuroinflammation* 17.

Mo, Y.S., Liu, B.J., Qiu, S., Wang, X.Q., Zhong, L.N., Han, X., and Mi, F.L. (2020). Down-regulation of microRNA-34c-5p alleviates neuropathic pain via the SIRT1/STAT3 signaling pathway in rat models of chronic constriction injury of sciatic nerve. *Journal Of Neurochemistry* 154**,** 301-315.

Neumann, E., Hermanns, H., Barthel, F., Werdehausen, R., and Brandenburger, T. (2015). Expression changes of microRNA-1 and its targets Connexin 43 and brain-derived neurotrophic factor in the peripheral nervous system of chronic neuropathic rats. *Molecular Pain* 11.

Pan, Z., Shan, Q., Gu, P., Wang, X.M., Tai, L.W., Sun, M., Luo, X., Sun, L., and Cheung, C.W. (2018). miRNA-23a/CXCR4 regulates neuropathic pain via directly targeting TXNIP/NLRP3 inflammasome axis. *Journal of Neuroinflammation* 15.

Pang, X., Tang, Y., and Zhang, D. (2016). Role of miR-145 in chronic constriction injury in rats. *Experimental and Therapeutic Medicine* 12**,** 4121-4127.

Peng, C.G., Li, L.L., Zhang, M.D., Gonzales, C.B., Parisien, M., Belfer, I., Usoskin, D., Abdo, H., Furlan, A., Haring, M., Lallemend, F., Harkany, T., Diatchenko, L., Hokfelt, T., Hjerling-Leffler, J., and Ernfors, P. (2017). miR-183 cluster scales mechanical pain sensitivity by regulating basal and neuropathic pain genes. *Science* 356**,** 1168-1171.

Qiu, S., Liu, B.J., Mo, Y.S., Wang, X.Q., Zhong, L.N., Han, X., and Mi, F.L. (2020). MiR-101 promotes pain hypersensitivity in rats with chronic constriction injury via the MKP-1 mediated MAPK pathway. *Journal Of Cellular And Molecular Medicine* 24**,** 8986-8997.

Sakai, A., Saitow, F., Maruyama, M., Miyake, N., Miyake, K., Shimada, T., Okada, T., and Suzuki, H. (2017). MicroRNA cluster miR-17-92 regulates multiple functionally related voltage-gated potassium channels in chronic neuropathic pain. *Nature Communications* 8.

Sakai, A., Saitow, F., Miyake, N., Miyake, K., Shimada, T., and Suzuki, H. (2013). miR-7a alleviates the maintenance of neuropathic pain through regulation of neuronal excitability. *Brain* 136**,** 2738-2750.

Sakai, A., and Suzuki, H. (2013). Nerve injury-induced upregulation of miR-21 in the primary sensory neurons contributes to neuropathic pain in rats. *Biochemical and Biophysical Research Communications* 435**,** 176-181.

Shao, J.P., Cao, J., Wang, J.N., Ren, X.H., Su, S.X., Li, M., Li, Z.H., Zhao, Q.Z., and Zang, W.D. (2016). MicroRNA-30b regulates expression of the sodium channel Nav1.7 in nerve injury-induced neuropathic pain in the rat. *Molecular Pain* 12.

Shi, D.N., Yuan, Y.T., Ye, D., Kang, L.M., Wen, J., and Chen, H.P. (2018a). MiR-183-5p alleviates chronic constriction injury-induced neuropathic pain through inhibition of TREK-1. *Neurochemical Research* 43**,** 1143-1149.

Shi, G.D., Shi, J.G., Liu, K., Liu, N., Wang, Y., Fu, Z.Y., Ding, J.D., Jia, L.S., and Yuan, W. (2013). Increased miR-195 aggravates neuropathic pain by inhibiting autophagy following peripheral nerve injury. *Glia* 61**,** 504-512.

Shi, J.S., Jiang, K., and Li, Z.D. (2018b). MiR-145 ameliorates neuropathic pain via inhibiting inflammatory responses and mTOR signaling pathway by targeting Akt3 in a rat model. *Neuroscience Research* 134**,** 10-17.

Simeoli, R., Montague, K., Jones, H.R., Castaldi, L., Chambers, D., Kelleher, J.H., Vacca, V., Pitcher, T., Grist, J., Al-Ahdal, H., Wong, L.F., Perretti, M., Lai, J., Mouritzen, P., Heppenstall, P., and Malcangio, M. (2017). Exosomal cargo including microRNA regulates sensory neuron to macrophage communication after nerve trauma. *Nature Communications* 8.

Su, S.X., Shao, J.P., Zhao, Q.Z., Ren, X.H., Cai, W.H., Li, L., Bai, Q., Chen, X.M., Xu, B., Wang, J., Cao, J., and Zang, W.D. (2017). MiR-30b Attenuates Neuropathic Pain by Regulating Voltage-Gated Sodium Channel Nav1.3 in Rats. *Frontiers In Molecular Neuroscience* 10.

Sun, L., Xia, R., Jiang, J., Wen, T., Huang, Z., Qian, R., Zhang, M.D., Zhou, M., and Peng, C. (2021). MicroRNA-96 is required to prevent allodynia by repressing voltage-gated sodium channels in spinal cord. *Progress in Neurobiology*.

Sun, W., Zhang, L., and Li, R. (2017). Overexpression of miR-206 ameliorates chronic constriction injury-induced neuropathic pain in rats via the MEK/ERK pathway by targeting brain-derived neurotrophic factor. *Neuroscience Letters* 646**,** 68-74.

Tan, M., Shen, L.L., and Hou, Y.Y. (2020). Epigenetic modification of BDNF mediates neuropathic pain via miR-30a-3p/EP300 axis in CCI rats. *Bioscience Reports* 40.

Tan, Y., Yang, J., Xiang, K., Tan, Q., and Guo, Q. (2015). Suppression of MicroRNA-155 Attenuates Neuropathic Pain by Regulating SOCS1 Signalling Pathway. *Neurochemical Research* 40**,** 550-560.

Tian, J., Song, T.Y., Wang, W.L., Wang, H., and Zhang, Z.W. (2020). miR-129-5p Alleviates Neuropathic Pain Through Regulating HMGB1 Expression in CCI Rat Models. *Journal Of Molecular Neuroscience* 70**,** 84-93.

Tozaki-Saitoh, H., Masuda, J., Kawada, R., Kojima, C., Yoneda, S., Masuda, T., Inoue, K., and Tsuda, M. (2019). Transcription factor MafB contributes to the activation of spinal microglia underlying neuropathic pain development. *GLIA* 67**,** 729-740.

Tramullas, M., Frances, R., De La Fuente, R., Velategui, S., Carcelen, M., Garcia, R., Llorca, J., and Hurle, M.A. (2018). MicroRNA-30c-5p modulates neuropathic pain in rodents. *Science Translational Medicine* 10.

Wan, L., Su, Z., Li, F., Gao, P., and Zhang, X. (2021). MiR-122-5p suppresses neuropathic pain development by targeting PDK4. *Neurochemical Research*.

Wang, C.H., Jiang, Q., Wang, M., and Li, D. (2015). MiR-19a targets suppressor of cytokine signaling 1 to modulate the progression of neuropathic pain. *International Journal Of Clinical And Experimental Pathology* 8**,** 10901-10907.

Wang, W., and Li, R. (2020). MiR-216a-5p alleviates chronic constriction injury-induced neuropathic pain in rats by targeting KDM3A and inactivating Wnt/β-catenin signaling pathway. *Neuroscience Research*.

Wang, X.H., Wang, H., Zhang, T., He, M., Liang, H., Wang, H., Xu, L.S., Chen, S., and Xu, M.H. (2019). Inhibition of MicroRNA-195 Alleviates Neuropathic Pain by Targeting Patched1 and Inhibiting SHH Signaling Pathway Activation. *Neurochemical Research* 44**,** 1690-1702.

Wang, Z., Liu, F., Wei, M., Qiu, Y., Ma, C., Shen, L., and Huang, Y. (2018). Chronic constriction injury-induced microRNA-146a-5p alleviates neuropathic pain through suppression of IRAK1/TRAF6 signaling pathway. *Journal of Neuroinflammation* 15.

Wen, J., He, T., Qi, F., and Chen, H. (2019). MiR-206-3p alleviates chronic constriction injury-induced neuropathic pain through targeting HDAC4. *Experimental Animals* 68**,** 213-220.

Xia, L., Zhang, Y.L., and Dong, T.L. (2016). Inhibition of MicroRNA-221 Alleviates Neuropathic Pain Through Targeting Suppressor of Cytokine Signaling 1. *Journal Of Molecular Neuroscience* 59**,** 411-420.

Xie, T., Zhang, J., Kang, Z., Liu, F., and Lin, Z. (2020). miR-101 down-regulates mTOR expression and attenuates neuropathic pain in chronic constriction injury rat models. *Neuroscience Research* 158**,** 30-36.

Xie, X., Ma, L., Xi, K., Zhang, W., and Fan, D. (2017). MicroRNA-183 Suppresses Neuropathic Pain and Expression of AMPA Receptors by Targeting mTOR/VEGF Signaling Pathway. *Cell Physiol Biochem* 41**,** 181-192.

Xu, B., Cao, J., Zhang, J., Jia, S.D., Wu, S.G., Mo, K., Wei, G.H., Liang, L.L., Miao, X.R., Bekker, A., and Tao, Y.X. (2017). Role of MicroRNA-143 in Nerve Injury-Induced Upregulation of Dnmt3a Expression in Primary Sensory Neurons. *Frontiers In Molecular Neuroscience* 10.

Xu, L., Wang, Q., Jiang, W., Yu, S., and Zhang, S. (2019). MiR-34c Ameliorates Neuropathic Pain by Targeting NLRP3 in a Mouse Model of Chronic Constriction Injury. *Neuroscience* 399**,** 125-134.

Yan, T., Zhang, F., Sun, C., Sun, J., Wang, Y., Xu, X., Shi, J., and Shi, G. (2018a). miR-32-5p-mediated Dusp5 downregulation contributes to neuropathic pain. *Biochemical and Biophysical Research Communications* 495**,** 506-511.

Yan, X.T., Ji, L.J., Wang, Z., Wu, X., Wang, Q., Sun, S., Lu, J.M., and Zhang, Y. (2017). MicroRNA-93 alleviates neuropathic pain through targeting signal transducer and activator of transcription 3. *International Immunopharmacology* 46**,** 156-162.

Yan, X.T., Zhao, Y., Cheng, X.L., He, X.H., Wang, Y., Zheng, W.Z., Chen, H., and Wang, Y.L. (2018b). Inhibition of miR-200b/miR-429 contributes to neuropathic pain development through targeting zinc finger E box binding protein-1. *Journal of Cellular Physiology* 233**,** 4815-4824.

Yang, F.R., Chen, J., Yi, H., Peng, L.Y., Hu, X.L., and Guo, Q.L. (2019). MicroRNA-7a ameliorates neuropathic pain in a rat model of spinal nerve ligation via the neurofilament light polypeptide-dependent signal transducer and activator of transcription signaling pathway. *Molecular Pain* 15.

Yang, Q., Liu, Z.H., and Chang, Y.L. (2016). Downregulation of miR-206 contributes to neuropathic pain in rats by enhancing RASA1 expression. *International Journal Of Clinical And Experimental Medicine* 9**,** 3146-3152.

Ye, G., Zhang, Y., Zhao, J., Chen, Y., Kong, L., Sheng, C., and Yuan, L. (2020). miR-384-5p ameliorates neuropathic pain by targeting SCN3A in a rat model of chronic constriction injury. *Neurological Research* 42**,** 299-307.

You, H.P., Zhang, L.H., Chen, Z.Y., Liu, W.F., Wang, H.G., and He, H.F. (2019). MiR-20b-5p relieves neuropathic pain by targeting Akt3 in a chronic constriction injury rat model. *Synapse* 73.

Zhan, L.Y., Lei, S.Q., Zhang, B.H., Li, W.L., Wang, H.X., Zhao, B., Cui, S.S., Ding, H., and Huang, Q.M. (2018). Overexpression of miR-381 relieves neuropathic pain development via targeting HMGB1 and CXCR4. *Biomedicine and Pharmacotherapy* 107**,** 818-823.

Zhang, J., Rong, L., Shao, J., Zhang, Y., Liu, Y., Zhao, S., Li, L., Yu, W., Zhang, M., Ren, X., Zhao, Q., and Zhu, C. (2021a). Epigenetic restoration of voltage-gated potassium channel Kv1.2 alleviates nerve injury-induced neuropathic pain. 156**,** 367-378.

Zhang, J., Zhang, H., and Zi, T. (2015). Overexpression of microRNA-141 relieves chronic constriction injury-induced neuropathic pain via targeting high-mobility group box 1. *International Journal of Molecular Medicine* 36**,** 1433-1439.

Zhang, X., Chen, Q., Shen, J., Wang, L., Cai, Y., and Zhu, K.R. (2020a). miR-194 relieve neuropathic pain and prevent neuroinflammation via targeting FOXA1. *Journal of Cellular Biochemistry* 121**,** 3278-3285.

Zhang, X., Guo, H., Xie, A., Liao, O., Ju, F., and Zhou, Y. (2020b). MicroRNA-144 relieves chronic constriction injury-induced neuropathic pain via targeting RASA1. *Biotechnology and Applied Biochemistry* 67**,** 294-302.

Zhang, X.L., Zhang, Y., Cai, W., Liu, Y., Liu, H.L., Zhang, Z.J., and Su, Z. (2020c). MicroRNA-128-3p Alleviates Neuropathic Pain Through Targeting ZEB1. *Neuroscience Letters* 729.

Zhang, Y., Liu, H.L., An, L.J., Li, L., Wei, M., Ge, D.J., and Su, Z. (2019a). miR-124-3p attenuates neuropathic pain induced by chronic sciatic nerve injury in rats via targeting EZH2. *Journal of Cellular Biochemistry* 120**,** 5747-5755.

Zhang, Y., Liu, J., Wang, X., Zhang, J., and Xie, C. (2021b). Extracellular vesicle-encapsulated microRNA-23a from dorsal root ganglia neurons binds to A20 and promotes inflammatory macrophage polarization following peripheral nerve injury. *Aging (Albany NY)* 13**,** 6752-6764.

Zhang, Y., Mou, J., Cao, L., Zhen, S., Huang, H., and Bao, H. (2018a). MicroRNA-142-3p relieves neuropathic pain by targeting high mobility group box 1. *International Journal of Molecular Medicine* 41**,** 501-510.

Zhang, Y., Su, Z., An, L.J., Li, L., Wei, M., Ge, D.J., and Liu, H.L. (2019b). miR-98 acts as an inhibitor in chronic constriction injury-induced neuropathic pain via downregulation of high-mobility group AT-hook 2. *Journal of Cellular Biochemistry* 120**,** 10363-10369.

Zhang, Y., Su, Z., Liu, H.L., Li, L., Wei, M., Ge, D.J., and Zhang, Z.J. (2018b). Effects of miR-26a-5p on neuropathic pain development by targeting MAPK6 in in CCI rat models. *Biomedicine and Pharmacotherapy* 107**,** 644-649.

Zhang, Z.J., Guo, J.S., Li, S.S., Wu, X.B., Cao, D.L., Jiang, B.C., Jing, P.B., Bai, X.Q., Li, C.H., Wu, Z.H., Lu, Y., and Gao, Y.J. (2018c). TLR8 and its endogenous ligand miR-21 contribute to neuropathic pain in murine DRG. *Journal of Experimental Medicine* 215**,** 3019-3037.

Zhong, L., Fu, K., Xiao, W., Wang, F., and Shen, L.L. (2019a). Overexpression of miR-98 attenuates neuropathic pain development via targeting STAT3 in CCI rat models. *Journal of Cellular Biochemistry* 120**,** 7989-7997.

Zhong, L., Xiao, W., Wang, F., Liu, J., and Zhi, L.J. (2019b). miR-21-5p inhibits neuropathic pain development via directly targeting C-C motif ligand 1 and tissue inhibitor of metalloproteinase-3. *Journal of Cellular Biochemistry* 120**,** 16614-16623.

Zhou, J., Zhuang, T., Ma, P., Shan, L.D., Sun, X.D., Gong, S., Tao, J., Yu, X.M., and Jiang, X.H. (2020). MicroRNA-547-5p-mediated interleukin-33/suppressor of tumorigenicity 2 signaling underlies the genesis and maintenance of neuropathic pain and is targeted by the therapy with bone marrow stromal cells. *Molecular Pain* 16.

Zhou, X., Zhang, C., Zhang, C., Peng, Y., Wang, Y., and Xu, H. (2017). MicroRNA-182-5p Regulates Nerve Injury-induced Nociceptive Hypersensitivity by Targeting Ephrin Type-b Receptor 1. *Anesthesiology* 126**,** 967-977.

Zhu, B., Gao, J., Ouyang, Y., Hu, Z., and Chen, X. (2019). Overexpression of mir138 ameliorates spared sciatic nerve injury-induced neuropathic pain through the anti-inflammatory response in mice. *Journal of Pain Research* 12**,** 3135-3145.

Zhu, H., Xue, C.B., Yao, M., Wang, H.K., Zhang, P., Qian, T.M., Zhou, S.L., Li, S.Y., Yu, B., Wang, Y.J., and Gu, X.S. (2018). miR-129 controls axonal regeneration via regulating insulin-like growth factor-1 in peripheral nerve injury. *Cell Death & Disease* 9.

Table S5. Experimentally verified miRNAs in drug-induced NP models

| **Article, Year** | **Country** | **Models** | **Animals** | **Region** | **miRNAs** | **Expression change** | **Target gene(s)** | **Functions** |
| --- | --- | --- | --- | --- | --- | --- | --- | --- |
| Huang et al., 2016 ([Huang et al., 2016](#_ENREF_2)) | China | paclitaxel-injected | rats | SDH | miR-500 | up | GAD67 | GABAergic synapses excitability |
| Ito et al., 2017 ([Ito et al., 2017](#_ENREF_3)) | Japan | oxaliplatin-induced | rats | L5 DRG | miR‐15b | up | BACE1 | Myelination |
| Li et al., 2019 ([Li et al., 2019](#_ENREF_4)) | China | oxaliplatin-induced | rats | L4-L6 DRGs | miR-30b-5p | down | Na_V_1.6 | Neuronal excitability |
| Miao et al., 2019 ([Miao et al., 2019](#_ENREF_5)) | China | oxaliplatin-induced | rats | L5-L6 SDH | miR-155 | up | TRPA1 | Neuroinflammation |
| Duan et al., 2020 ([Duan et al., 2020](#_ENREF_1)) | China | bortezomib-induced | rats | L5-L6 SDH | miR-155 | up | TNFR1, p38-MAPK, JNK, TRPA1 | Neuroinflammation |
| Zhang et al., 2021 ([Zhang and Chen, 2021](#_ENREF_6)) | China | oxaliplatin-induced | rats | DRG | miR-141-5p | down | TRPA1 | Neuroinflammation |

Abbreviations: BACE1: aspartyl protease β-site amyloid precursor protein-cleaving enzyme; DRG: dorsal root ganglion; GAD: GABAergic synapses excitability; Na_V_: voltage-gated sodium channel; SDH: spinal dorsal horn; TNFR1: tumor necrosis factor-α receptor; TRPA1: transient receptor potential ankyrin 1.

References

Duan, Z.S., Zhang, J., Li, J., Pang, X.C., and Wang, H.S. (2020). Inhibition of microRNA-155 Reduces Neuropathic Pain During Chemotherapeutic Bortezomib via Engagement of Neuroinflammation. *Frontiers In Oncology* 10.

Huang, Z.Z., Wei, J.Y., Ou-Yang, H.D., Li, D., Xu, T., Wu, S.L., Zhang, X.L., Liu, C.C., Ma, C., and Xin, W.J. (2016). Mir-500-mediated GAD67 downregulation contributes to neuropathic pain. *Journal of Neuroscience* 36**,** 6321-6331.

Ito, N., Sakai, A., Miyake, N., Maruyama, M., Iwasaki, H., Miyake, K., Okada, T., Sakamoto, A., and Suzuki, H. (2017). miR-15b mediates oxaliplatin-induced chronic neuropathic pain through BACE1 down-regulation. *British Journal of Pharmacology* 174**,** 386-395.

Li, L., Shao, J., Wang, J., Liu, Y., Zhang, Y., Zhang, M., Zhang, J., Ren, X., Su, S., Li, Y., Cao, J., and Zang, W. (2019). MiR-30b-5p attenuates oxaliplatin-induced peripheral neuropathic pain through the voltage-gated sodium channel Nav1.6 in rats. *Neuropharmacology* 153**,** 111-120.

Miao, F.H., Wang, R., Cui, G.Z., Li, X.G., Wang, T., and Li, X. (2019). Engagement of MicroRNA-155 in Exaggerated Oxidative Stress Signal and TRPA1 in the Dorsal Horn of the Spinal Cord and Neuropathic Pain During Chemotherapeutic Oxaliplatin. *Neurotoxicity Research* 36**,** 712-723.

Zhang, H., and Chen, H. (2021). TRPA1 involved in miR-141-5p-alleviated neuropathic pain induced by oxaliplatin. *NeuroReport***,** 284-290.

Table 5. Experimentally verified miRNAs in disease-induced NP models

| **Article, Year** | **Country** | **Models** | **Animals** | **Region** | **miRNAs** | **Expression change** | **Target gene** | **Functions** |
| --- | --- | --- | --- | --- | --- | --- | --- | --- |
| Elramah et al., 2017 ([Elramah et al., 2017](#_ENREF_3)) | France | bone cancer pain | mice | SDH | miR-124 | down | Synpo | Synaptic transmission |
| Gandla et al., 2017 ([Gandla et al., 2017](#_ENREF_5)) | Germany | bone metastatic pain | mice | DRG | miR-34c-5p | up | Cav2.3 | Neuronal excitability |
| Yang et al., 2017 ([Yang et al., 2017](#_ENREF_11)) | China | STZ-injected DNP model | mice | SDH | miR-190a-5p | down | SLC17A6 | Neuroinflammation |
| Feng et al., 2018 ([Feng et al., 2018](#_ENREF_4)) | China | STZ-injected DNP model | rats | sciatic nerves | miR-146a | down | NF-κB | Neuroinflammation |
| Chen et al., 2019 ([Chen et al., 2019](#_ENREF_2)) | China | STZ-injected DNP model | rats | sciatic nerves | miR-155 | up | Nrf2 | Neuroinflammation |
| Wu et al., 2019 ([Wu et al., 2019a](#_ENREF_7)) | China | STZ-injected DNP model | mice | SDH | miR-193a | down | HMGB1 | Neuroinflammation |
| Wu et al., 2019 ([Wu et al., 2019b](#_ENREF_8)) | China | bone cancer pain | mice | spinal cord | miR-329 | down | LPAR1 | Neuroinflammation |
| Chang et al., 2020 ([Chang et al., 2020](#_ENREF_1)) | China | STZ-injected DNP model | rats | sciatic nerve, RSC96 Schwann cells | miR-133a-3p | up | p-p38 MAPK | Neuroimmune |
| Liu et al., 2020 ([Liu et al., 2020](#_ENREF_6)) | China | bone cancer pain | rats | L4-L6 spinal cords | miR-300 | down | HMGB1 | Neuroinflammation |
| Yan et al., 2020 ([Yan et al., 2020](#_ENREF_10)) | China | STZ-injected DNP model | rats | L3-L5 DRGs, DRG neurons | miR-145 | down | Na_V_1.8 | Neuroinflammation |
| Wu et al., 2020 ([Wu et al., 2020](#_ENREF_9)) | China | spontaneous DNP | mice | DRG | miR-590-3p | down | RAP1A | T cells proliferation and migration |

Abbreviations: DNP: diabetic neuropathic pain; DRG: dorsal root ganglion; HMGB1: high mobility group box 1 protein; LPAR1: lysophosphatidic acid receptor 1; Na_V_: voltage-gated sodium channel; NF-κB: nuclear factor kappa-light-chain-enhancer of activated B cells; RAP1A: Ras-associated protein 1A; SDH: spinal dorsal horn; STZ: streptozocin; Synpo: synaptopodin.

References

Chang, L.L., Wang, H.C., Tseng, K.Y., Su, M.P., Wang, J.Y., Chuang, Y.T., Wang, Y.H., and Cheng, K.I. (2020). Upregulation of miR-133a-3p in the Sciatic Nerve Contributes to Neuropathic Pain Development. *Molecular Neurobiology* 57**,** 3931-3942.

Chen, J., Li, C., Liu, W., Yan, B., Hu, X., and Yang, F. (2019). miRNA-155 silencing reduces sciatic nerve injury in diabetic peripheral neuropathy. *Journal of Molecular Endocrinology* 63**,** 227-238.

Elramah, S., Lopez-Gonzalez, M.J., Bastide, M., Dixmerias, F., Roca-Lapirot, O., Wielanek-Bachelet, A.C., Vital, A., Leste-Lasserre, T., Brochard, A., Landry, M., and Favereaux, A. (2017). Spinal miRNA-124 regulates synaptopodin and nociception in an animal model of bone cancer pain. *Scientific Reports* 7.

Feng, Y., Chen, L., Luo, Q., Wu, M., Chen, Y., and Shi, X. (2018). Involvement of microRNA-146a in diabetic peripheral neuropathy through the regulation of inflammation. *Drug Des Devel Ther* 12**,** 171-177.

Gandla, J., Lomada, S.K., Lu, J.N., Kuner, R., and Bali, K.K. (2017). miR-34c-5p functions as pronociceptive microRNA in cancer pain by targeting Cav2.3 containing calcium channels. *Pain* 158**,** 1765-1779.

Liu, C., Yang, J., Liu, H., Xia, T., and Zhang, F. (2020). miR-300 mitigates cancer-induced bone pain through targeting HMGB1 in rat models. *Genes and Genomics* 42**,** 309-316.

Wu, B., Guo, Y.Y., Chen, Q.B., Xiong, Q.J., and Min, S. (2019a). MicroRNA-193a Downregulates HMGB1 to Alleviate Diabetic Neuropathic Pain in a Mouse Model. *Neuroimmunomodulation* 26**,** 250-257.

Wu, X.P., Yang, Y.P., She, R.X., Xing, Z.M., Chen, H.W., and Zhang, Y.W. (2019b). microRNA-329 reduces bone cancer pain through the LPAR1-dependent LPAR1/ERK signal transduction pathway in mice. *Therapeutic Advances In Medical Oncology* 11.

Wu, Y., Gu, Y., and Shi, B. (2020). miR-590-3p Alleviates diabetic peripheral neuropathic pain by targeting RAP1A and suppressing infiltration by the T cells. *Acta biochimica Polonica* 67**,** 587-593.

Yan, J., Yu, H., Shen, J., Han, C., Li, C., Shen, X., and Li, B. (2020). Early Over-Expressing of microRNA-145 Effectively Precludes the Development of Neuropathic Mechanical Hyperalgesia via Suppressing Nav1.8 in Diabetic Rats. *Pain Physician* 23**,** E673-e686.

Yang, D., Yang, Q., Wei, X., Liu, Y., Ma, D., Li, J., Wan, Y., and Luo, Y. (2017). The role of miR-190a-5p contributes to diabetic neuropathic pain via targeting SLC17A6. *Journal of Pain Research* 10**,** 2395-2403.

## Supplementary Figures


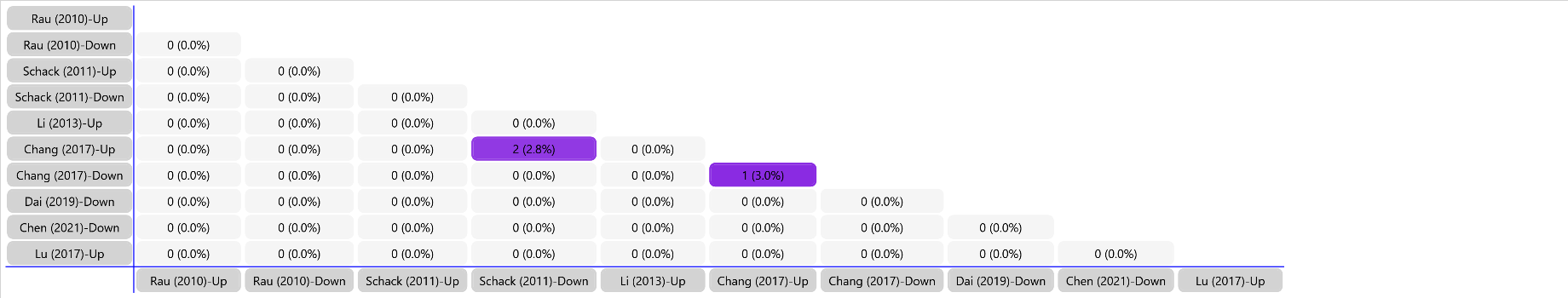


**Figure S1.** Matrix table analysis for miRNAs expression profiles in dorsal root ganglion of NP surgical models. The number and percentage of co-regulated miRNAs were highlighted. Overlapping miRNAs: 2 (2.8%): miR-21, miR-221; 1 (3.0%): miR-466c.


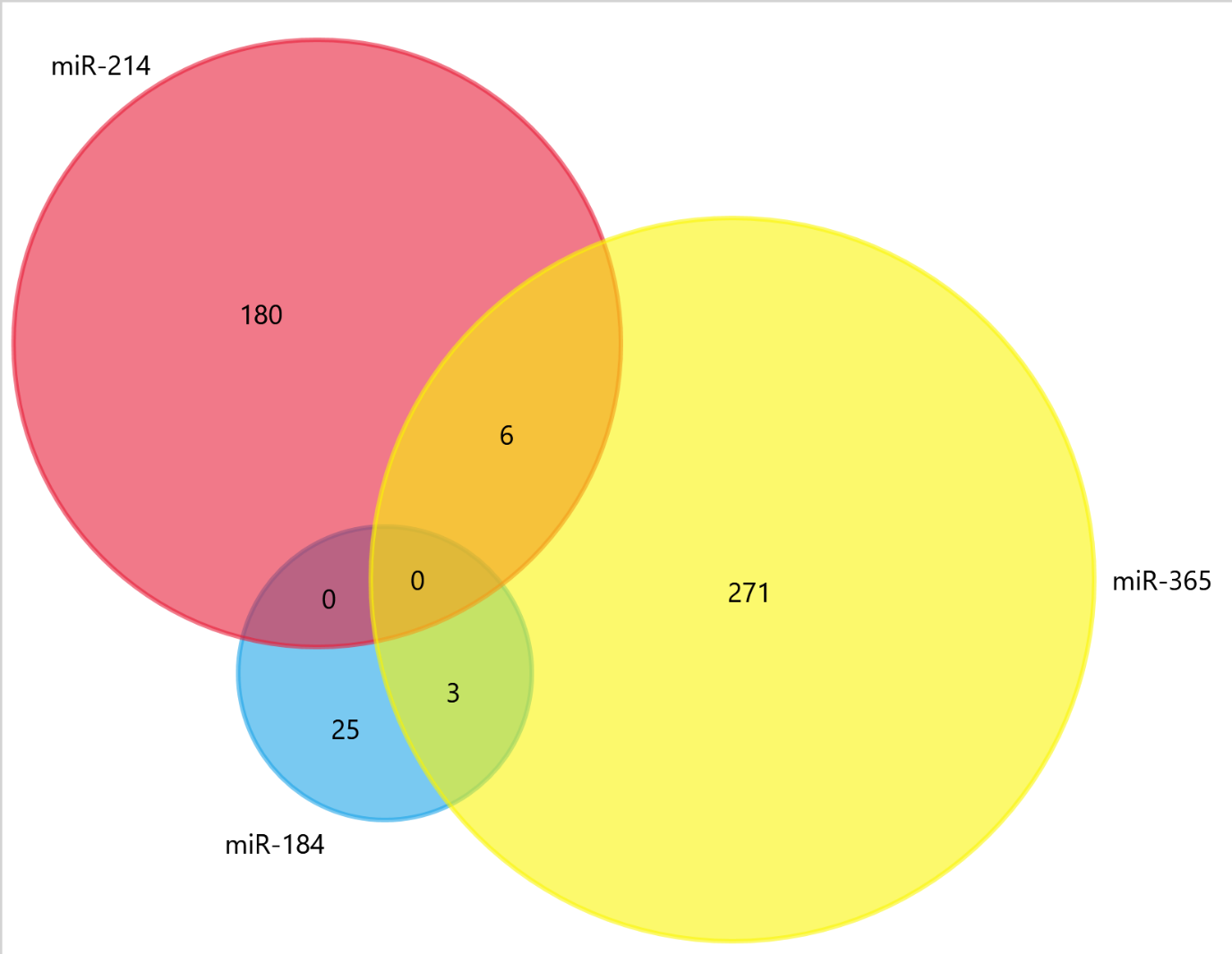


**Figure S2.** Venn diagram analysis. Overlapping target genes of miR-365, miR-184 and miR-214 in spinal cords. These three down-regulated genes were showed in Table S2 and have been observed in two or more studies.


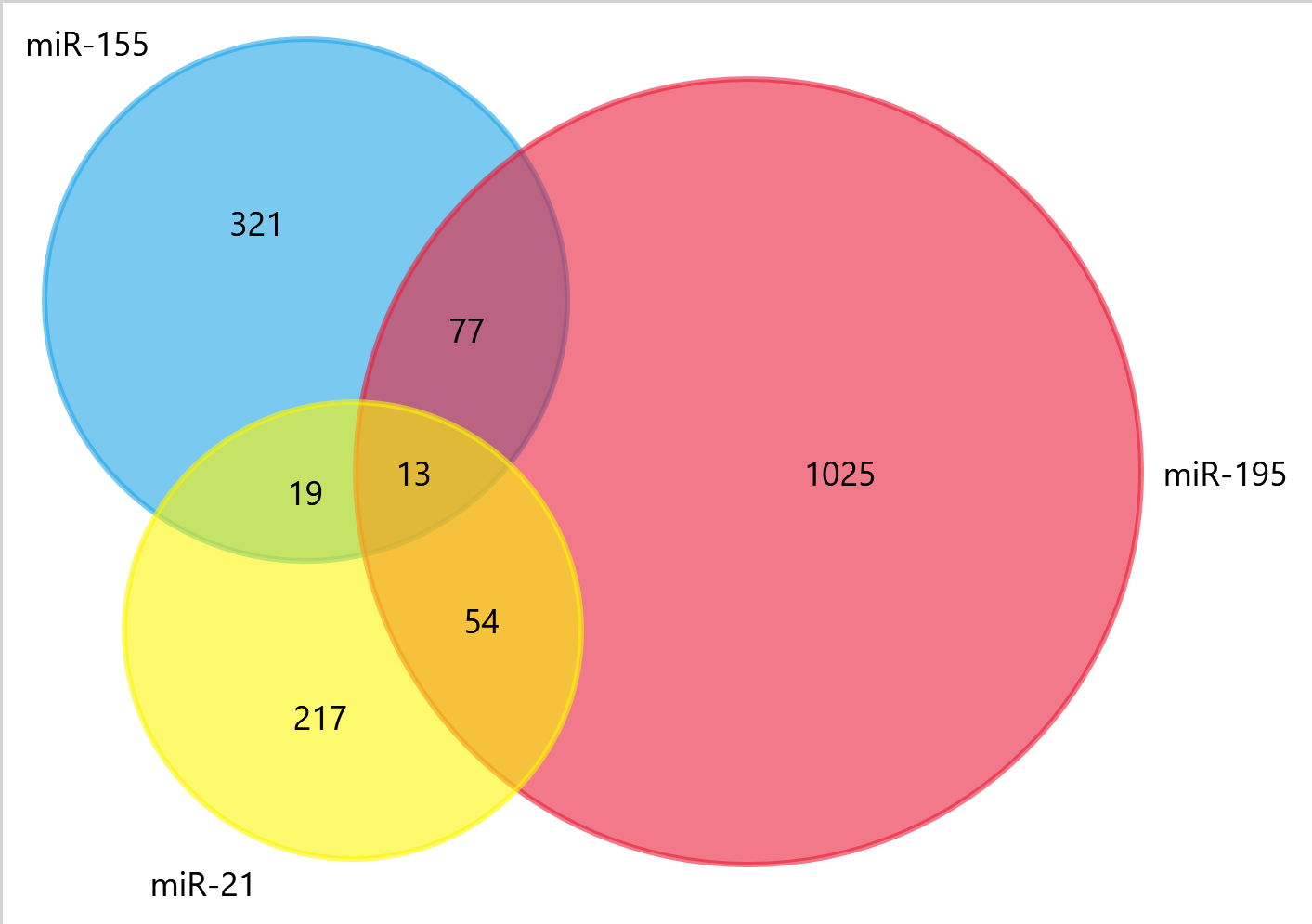


**Figure S3.** Venn diagram analysis. Overlapping target genes of miR-155, miR-21 and miR-195 in dorsal root ganglions. These three up-regulated genes were showed in Table S4 and have been observed in two or more experimentally verified studies.

s


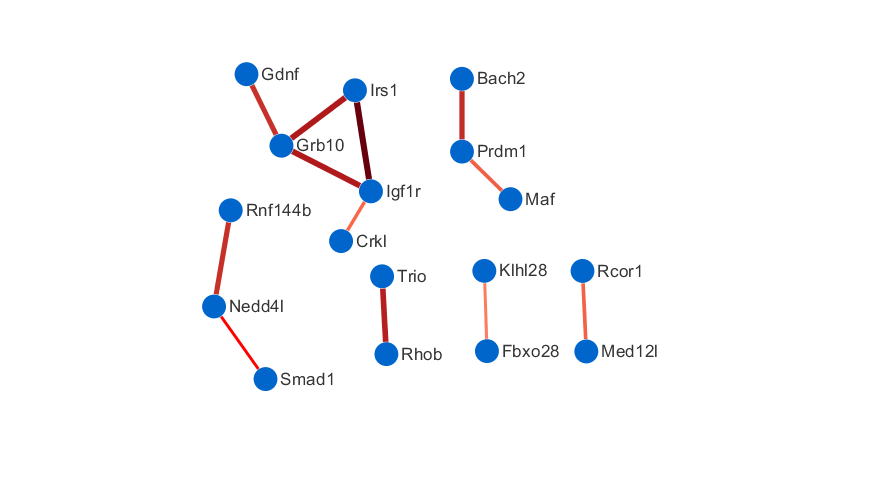


**Figure S4.** Protein-protein interaction (PPI) analysis. 17 genes showed interactions in 63 overlapping target genes of miR-183 cluster, miR-30b and miR-145. Large sizes and dark colors of edges meant high value of combined scores. High confidence score of 0.7 was selected to construction PPI network.
